# Supplementary material for: Elucidation of interactions regulating conformational stability and dynamics of SARS-CoV-2 S-protein
Source: Biophys J. 2021 Jan 21;120(6):1060–71. doi: 10.1016/j.bpj.2021.01.012 (PMC7825899; doi:10.1016/j.bpj.2021.01.012)
Supplement: Document S1. Figs. S1–S10 [file mmc1.pdf]

Biophysical Journal, Volume *120*

Supplemental information

Elucidation of interactions regulating conformational stability and dynamics of SARS-CoV-2 S-protein

Takaharu Mori, Jaewoon Jung, Chigusa Kobayashi, Hisham M. Dokainish, Suyong Re, and Yuji Sugita

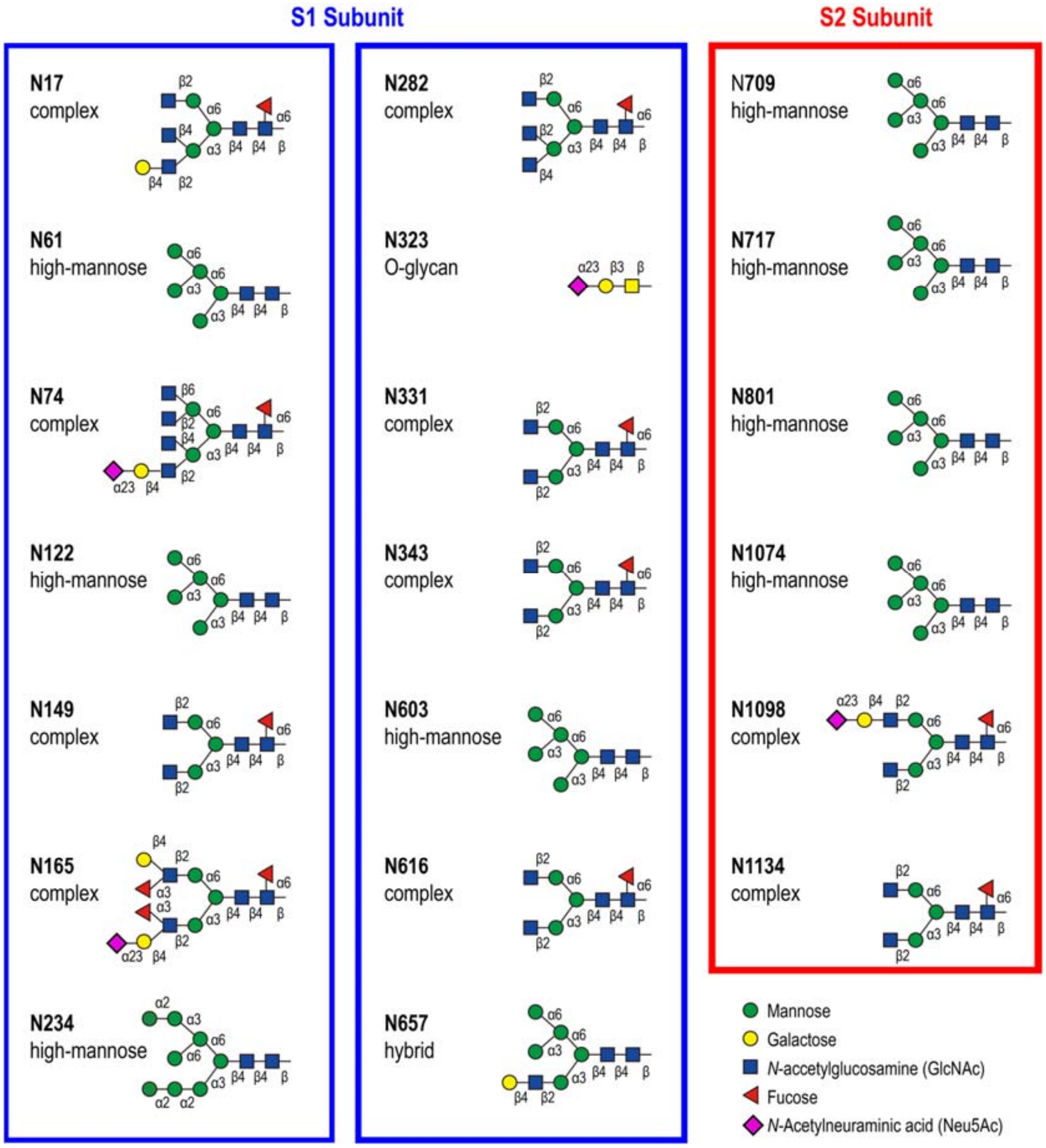

Figure S1. Summary of glycan structures and types attached to the S1 and S2 subunits of the S-protein used in all-the current all-atom MD simulations.

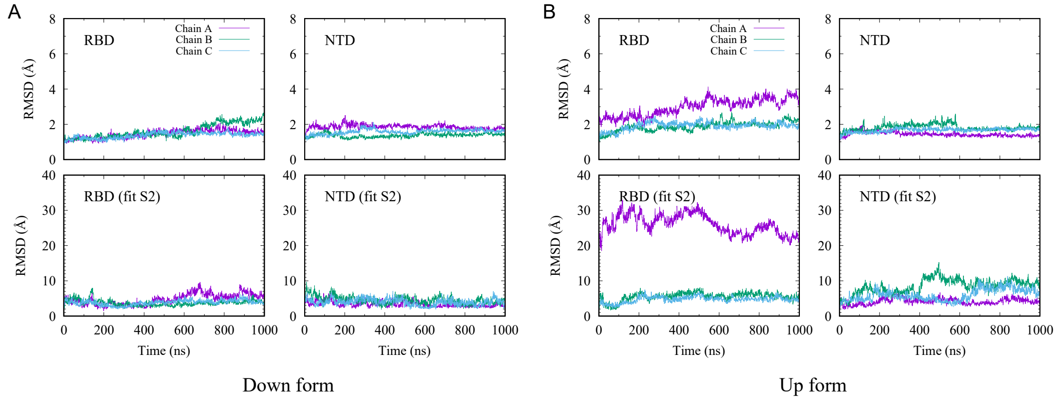

Figure S2. Root-mean-square deviations (RMSDs) of RBD and NTD in S-protein obtained in MD1_Down (A) and MD1_Up (B). In each graph, domains in chains A, B, and C are shown in purple, green, and light blue. In the calculations of RMSD, the superimposed regions in top and bottom are RBD/NTD itself and the S2 subunit in the Down form, respectively.

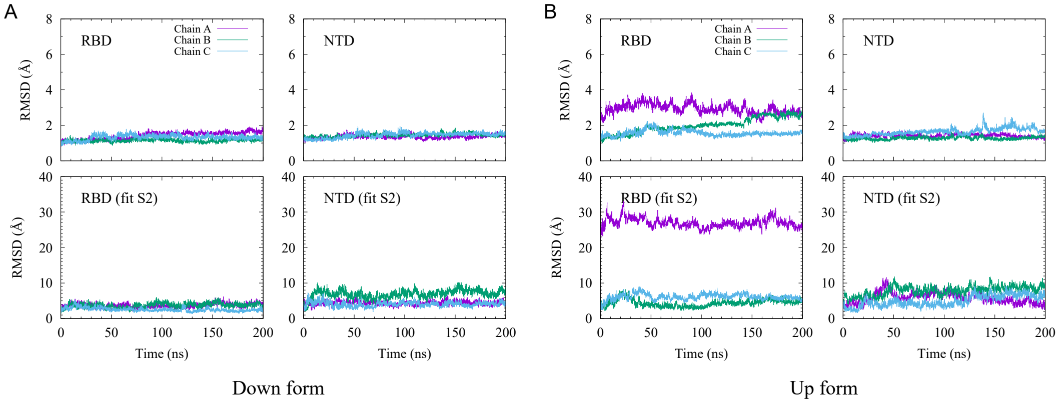

Figure S3. Root-mean-square deviations (RMSDs) of RBD and NTD in S-protein obtained in MD2_Down (A) and MD2_Up (B). In each graph, domains in chains A, B, and C are shown in purple, green, and light blue. In the calculations of RMSD, the superimposed regions in top and bottom are RBD/NTD itself and the S2 subunit in the Down form, respectively.

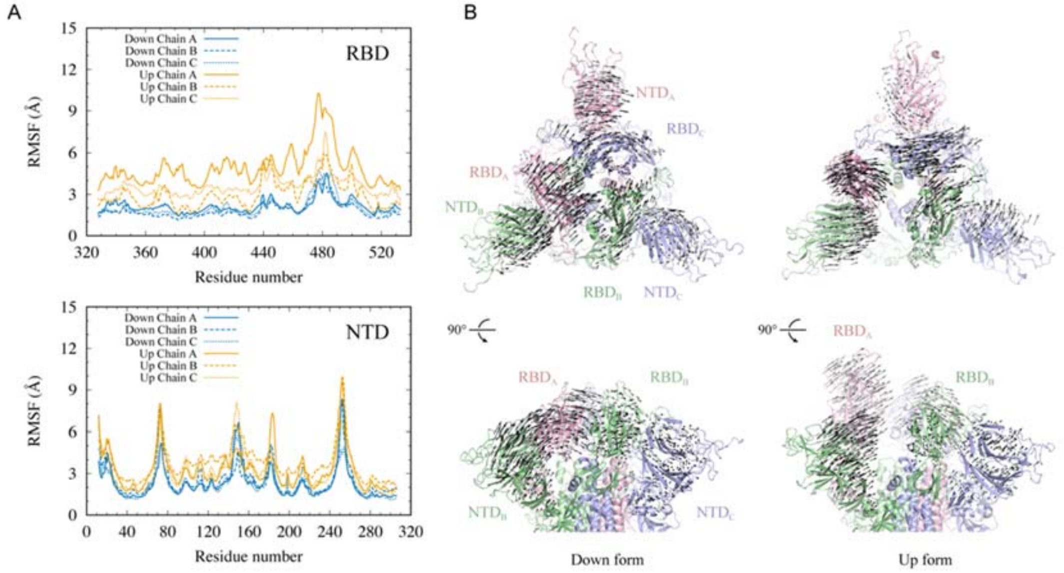

Figure S4. (A) Root-mean-square fluctuations (RMSFs) of the Cα atoms in residues belong to RBD (top) and NTD (bottom) in MD2_Down and MD2_Up. The lowest mode in principal component analysis (PCA) of the simulation trajectories of Down (left) and Up (right) forms. For clarity, the vectors are magnified 100 times. Both top and side views are shown in top and bottom figures, respectively. To obtain the RMSF and PCA, the S2 subunit of simulation snapshots was superimposed to that in Down form of S-protein.

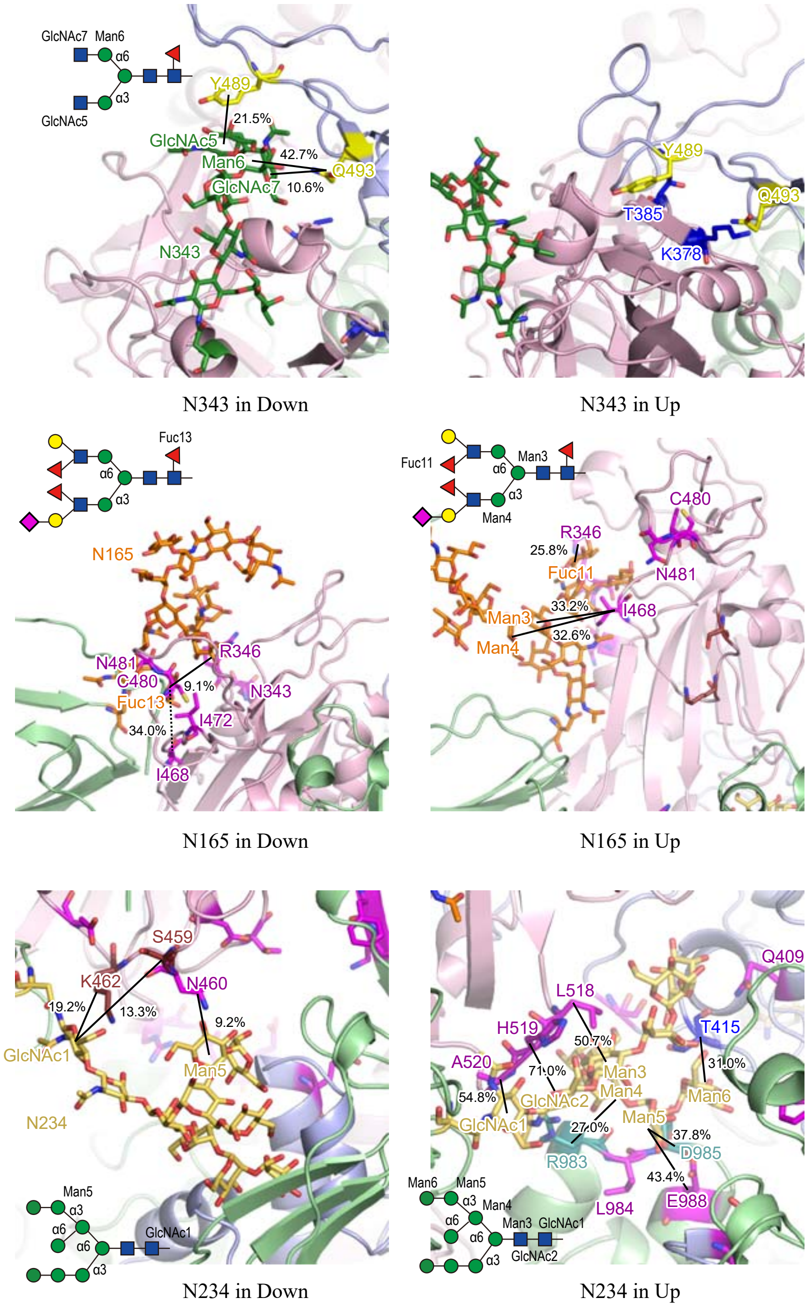

Figure S5. Interactions between amino acids and glycans at N343, N165, and N234. The probabilities of the contact formation in the last 500 ns of MD1_Down/Up are shown.

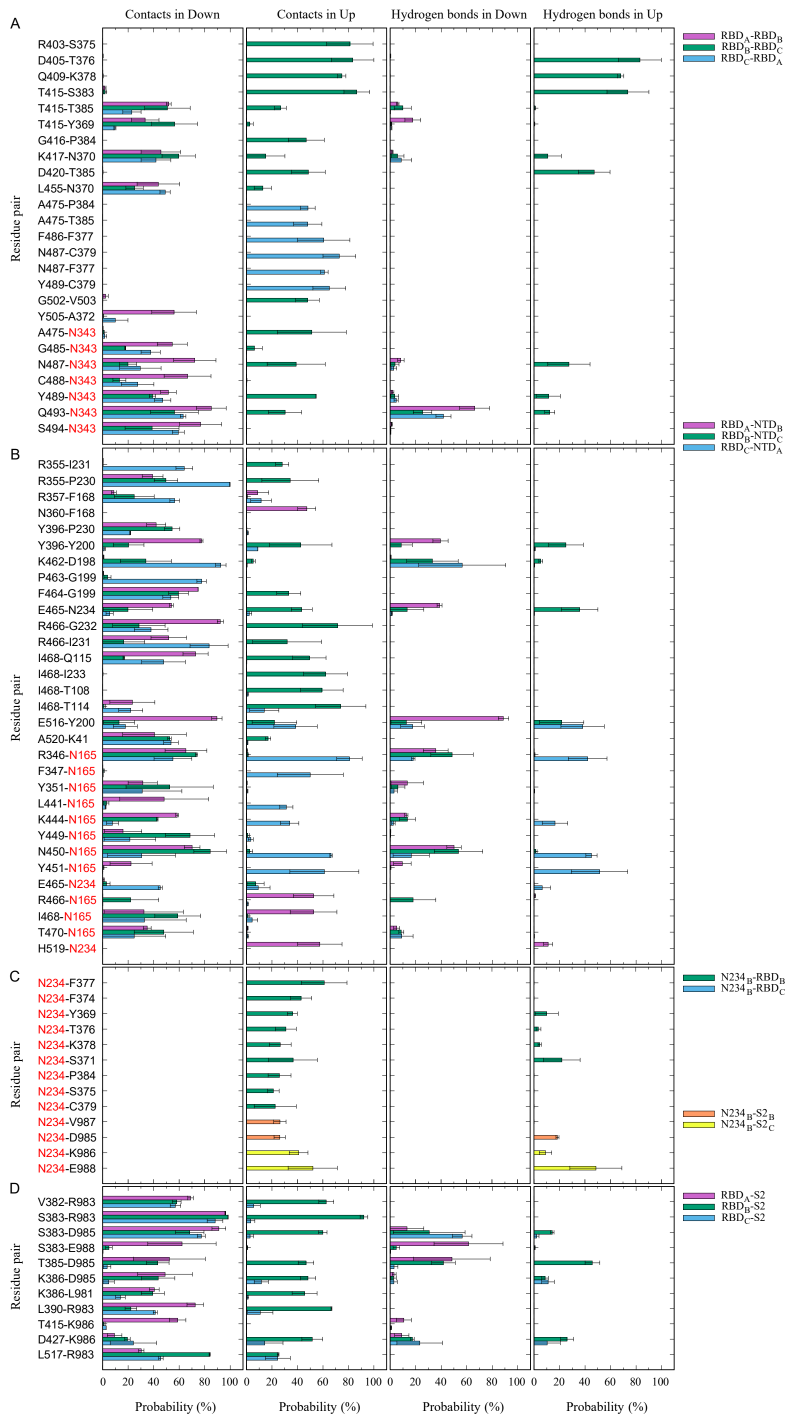

Figure S6. The interdomain contacts and hydrogen bonds in MD2_Down and MD2_Up. (A) Those interactions between different RBDs: RBD_A-RBD_B (purple), RBD_B-RBD_C (green), RBD_C-RBD_A

(light blue), (B) those between RBD and NTD: RBD_A-NTD_B (purple), RBD_B-NTD_C (green), RBD_C-NTD_A (light blue), (C) those between N234 and RBDs/S2: $N234_B$-RBD_B (green), $N234_B$-RBD_C (light blue), $N234_B$-$S2_B$ (orange), $N234_B$-$S2_C$ (yellow), (D) those between RBD and S2: RBD_A-S2 (purple), RBD_B-S2 (green), RBD_C-S2 (light blue). Red characters mean the glycosylated amino-acid residues. In the analysis, a contact is defined when the minimum residue distance is shorter than 2.5 Å. A hydrogen bond is decided if the D…A (donor…acceptor) distance is shorter than 3.4 Å, the DH…A angle is smaller than 120°, and HD…A angle is greater than 30°. A 200-ns trajectory is divided into two 100-ns trajectories and the average numbers of contacts and hydrogen bonds are shown as bars. The maximum and minimum numbers are shown in error bars. Only the residue pairs that have more than 45% (for panels A, B, and D) or 20 % (for panel C) of contacts in either MD2_Down or MD2_Up are shown.

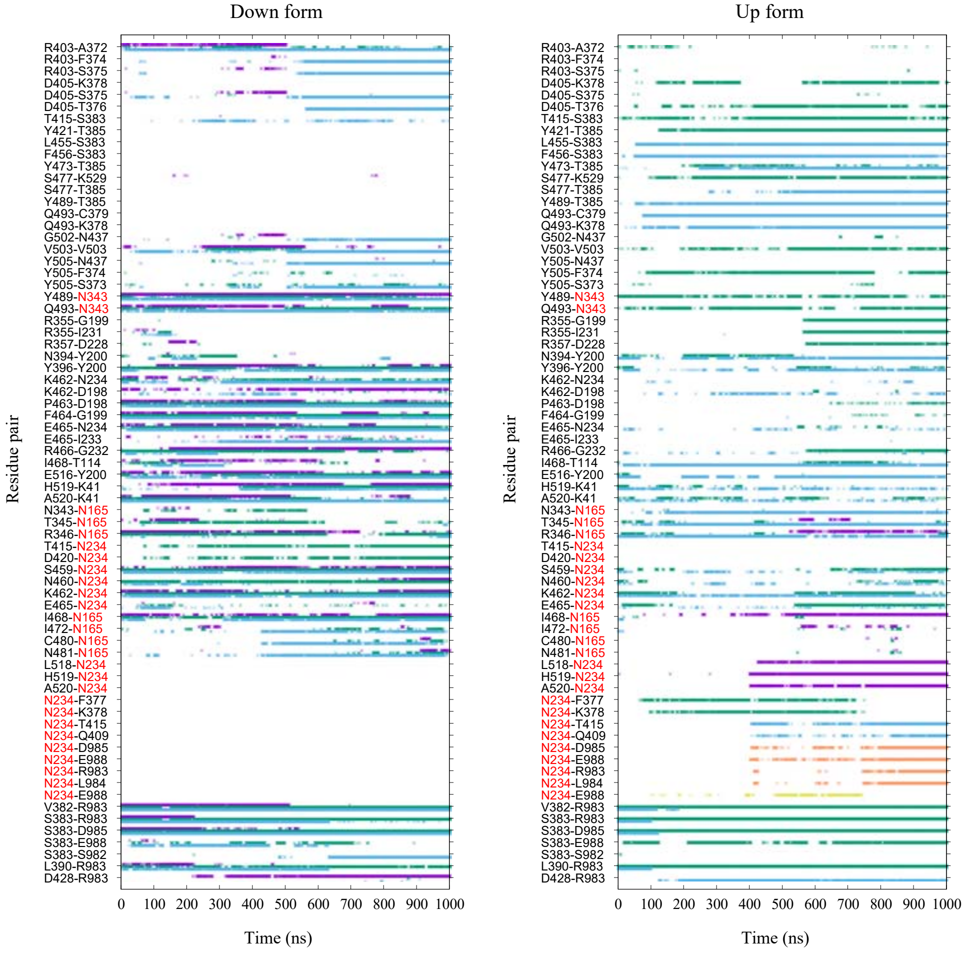

Figure S7. Time series of inter-domain contacts in MD1_Down (left) and MD1_Up (right). The color definition for dots is same as in Figure 3**.** Black characters mean the amino-acid residues, and red characters mean the glycan part of the glycosylated amino-acid residues.

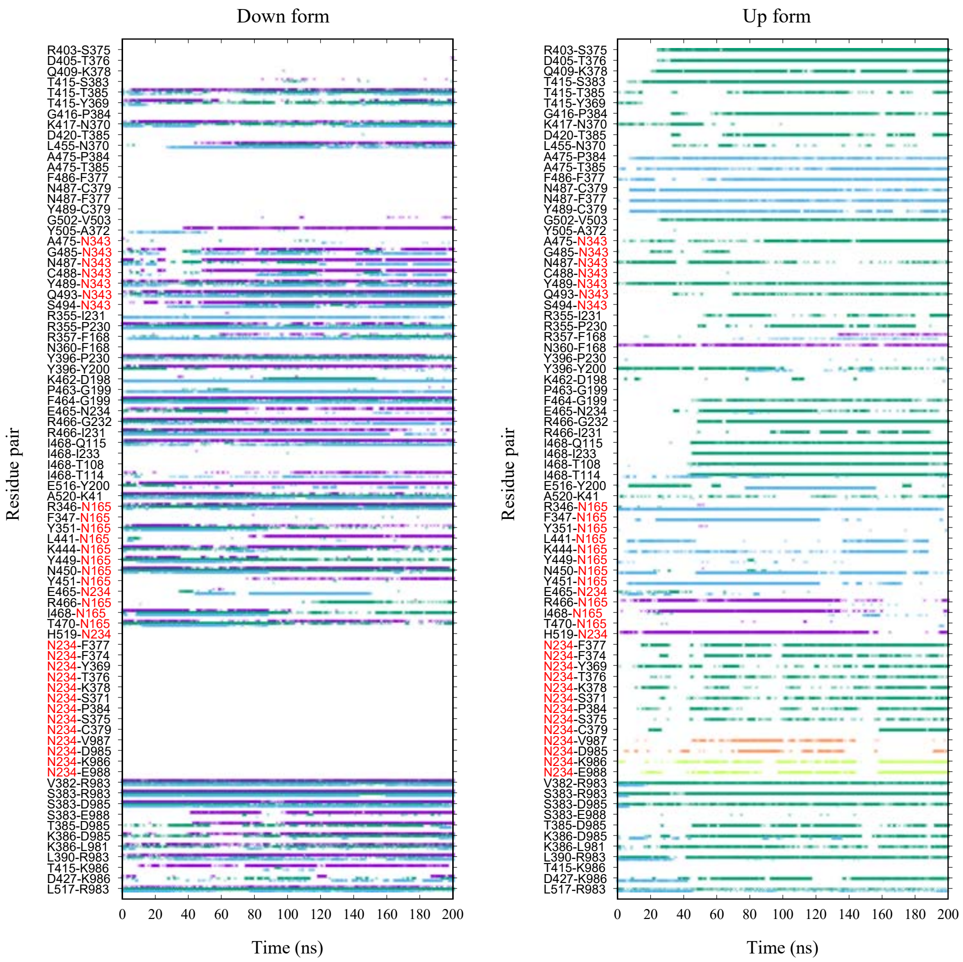

Figure S8. Time series of inter-domain contacts in MD2_Down (left) and MD2_Up (right). The color definition for dots is same as in Figure S5**.** Black characters mean the amino-acid residues, and red characters mean the glycan part of the glycosylated amino-acid residues.

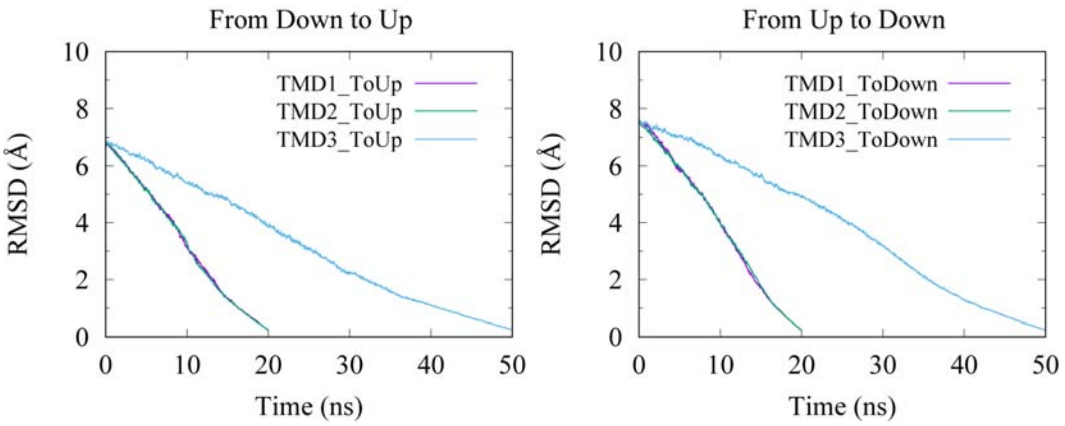

Figure S9. Time series of RMSD in TMD simulations from Down to Up (left) and from Up to Down (right). The RMSD was calculated using the Cα atoms of S1 and S2 subunits in the cryo-EM structure of the Up (PDB: 6VSB) or Down form (PDB: 6VXX).

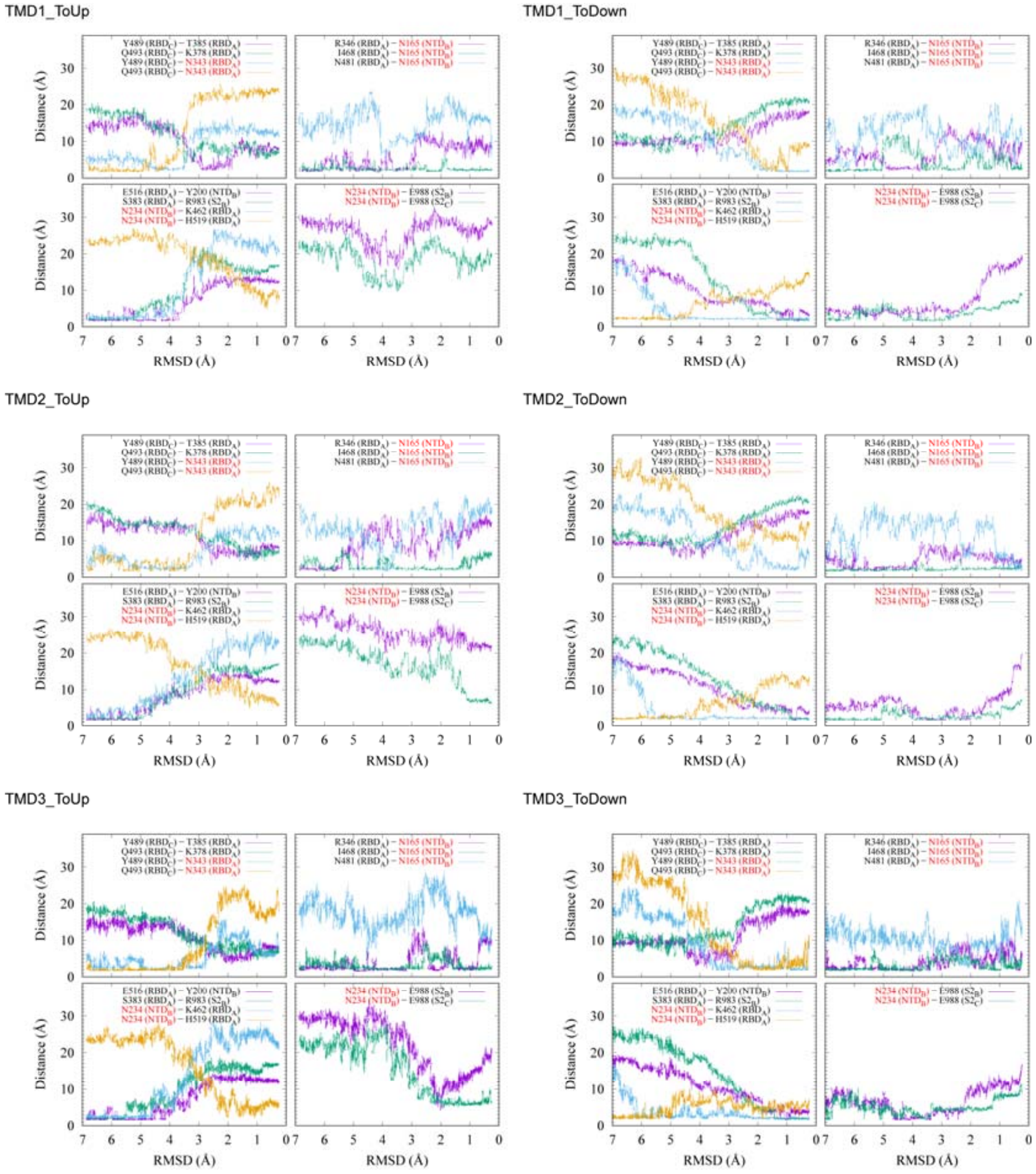

Figure S10. The minimum distances of the inter-domain amino-acid residue pairs and residue-glycan pairs in the three TMD_ToUp (left panels) and three TMD_ToDown (right panels) simulations. Red characters mean the glycosylated amino-acid residues. RMSD in TMD simulations is measured using the cryo-EM structure in the Up or Down form as a reference.
