# Supplementary material for: Elucidation of interactions regulating conformational stability and dynamics of SARS-CoV-2 S-protein
Source: Biophys J. 2021 Jan 21;120(6):1060–71. doi: 10.1016/j.bpj.2021.01.012 (PMC7825899; doi:10.1016/j.bpj.2021.01.012)
Supplement: Document S2. Article plus supporting material [file mmc2.pdf]

Biophysical Journal
Article

Elucidation of interactions regulating conformational stability and dynamics of SARS-CoV-2 S-protein

Takaharu Mori,[1] Jaewoon Jung,[1,2] Chigusa Kobayashi,[2] Hisham M. Dokainish,[1] Suyong Re,[3,4] and Yuji Sugita[1,2,3,*]

[1]Theoretical Molecular Science Laboratory, RIKEN Cluster for Pioneering Research, Wako, Japan; [2]Computational Biophysics Research Team, RIKEN Center for Computational Science, Kobe, Japan; [3]Laboratory for Biomolecular Function Simulation, RIKEN Center for Biosystems Dynamics Research, Kobe, Japan; and [4]Center for Drug Design Research, National Institutes of Biomedical Innovation, Health, and Nutrition, Osaka, Japan

ABSTRACT The ongoing COVID-19 pandemic caused by the new coronavirus, SARS-CoV-2, calls for urgent developments of vaccines and antiviral drugs. The spike protein of SARS-CoV-2 (S-protein), which consists of trimeric polypeptide chains with glycosylated residues on the surface, triggers the virus entry into a host cell. Extensive structural and functional studies on this protein have rapidly advanced our understanding of the S-protein structure at atomic resolutions, although most of these structural studies overlook the effect of glycans attached to the S-protein on the conformational stability and functional motions between the inactive down and active up forms. Here, we performed all-atom molecular dynamics simulations of both down and up forms of a fully glycosylated S-protein in solution as well as targeted molecular dynamics simulations between them to elucidate key interdomain interactions for stabilizing each form and inducing the large-scale conformational transitions. The residue-level interaction analysis of the simulation trajectories detects distinct amino acid residues and N-glycans as determinants on conformational stability of each form. During the conformational transitions between them, interdomain interactions mediated by glycosylated residues are switched to play key roles on the stabilization of another form. Electrostatic interactions, as well as hydrogen bonds between the three receptor binding domains, work as driving forces to initiate the conformational transitions toward the active form. This study sheds light on the mechanisms underlying conformational stability and functional motions of the S-protein, which are relevant for vaccine and antiviral drug developments.

SIGNIFICANCE Pandemic viral infections have posed a threat to humankind from time to time and encourage development of vaccines or antiviral drugs based on structural and functional information of proteins in target viruses. X-ray and cryo-electron microscopy structures of virus proteins provide atomistically detailed information while leaving much of their dynamic aspects, including the role of glycosylated residues, elusive. Using all-atom molecular dynamics simulations, we characterize the conformational fluctuations and interdomain interactions of SARS-CoV-2 spike protein. The interdomain hydrogen-bond and glycan interactions are rearranged in the conformational transition from the inactive to the active forms, which is responsible for better understanding of molecular mechanisms underlying virus entry into a host cell and the rational designs of vaccines and antiviral drugs.

INTRODUCTION

Severe acute respiratory syndrome coronavirus 2 (SARS-CoV-2) has caused coronavirus disease 2019 (COVID-19) (1–3). The rapid spreading of this virus infection since December of 2019 has driven researchers in both academic institutes and pharmaceutical companies to develop vaccines and antiviral drugs to meet demand as fast as possible (4,5). SARS-CoV-2 is an enveloped RNA virus belonging to the family Coronaviridae. The spike protein (S-protein) protruding from the envelope mediates the virus entry into a host cell, and therefore, it is one of the primary targets for vaccine and antiviral drug developments. The S-protein is a trimeric protein, and each monomer consists of S1 and S2 subunits responsible for host-cell receptor binding and

Submitted September 15, 2020, and accepted for publication January 13, 2021.

*Correspondence: sugita@riken.jp

Editor: Tamar Schlick.

https://doi.org/10.1016/j.bpj.2021.01.012

© 2021 Biophysical Society.

This is an open access article under the CC BY license (http://creativecommons.org/licenses/by/4.0/).

membrane fusion, respectively (6–8). Sequence similarity between SARS-CoV and SARS-CoV-2 S-proteins (sharing 76% amino acid sequence) (3,9,10) helps to prompt screening of already approved drugs by repositioning and to design an effective vaccine, but no prominent success has been reported yet, to our knowledge.

Structural information of a protein targeted in vaccines and antiviral drugs developments is generally essential. Until now, a large number of high-resolution x-ray and cryo-electron microscopy (cryo-EM) structures of SARS-CoV-2 proteins have been accumulated rapidly in the RCSB Protein Data Bank (PDB) (11). 369 structures have been deposited to the “COVID-19/SARS-CoV-2 Resources” in the PDB as of September 4, 2020. They include S-proteins both at the prefusion and postfusion states (9,12–16) and S-protein bound to the peptidase domain of angiotensin-converting enzyme 2 (ACE2) receptor (17–21). Cryo-EM structures show that the S-protein at prefusion state takes at least two possible conformations: the down form with three receptor-binding domains (RBDs) buried at the interface and the up form with one of the RBDs protruding from the interface (9). The atomic structures of S-protein bound to human ACE2 receptor indicate that the up form plays a prominent role in binding (18,22). Furthermore, the cryo-EM structure of a human antibody bound to the SARS-CoV-2 S-protein suggests that more than one RBD can take up forms to bind a variable domain of antibody individually (17). The highly dynamic nature of the S-protein, in particular its RBD, is responsible for the entry process of SARS-CoV-2 into a host cell.

Another key feature of SARS-CoV-2 S-protein is that it is highly glycosylated like other viral envelope proteins, such as human immunodeficiency virus 1 and influenza (23). Mass spectroscopy of the SARS-CoV-2 S-protein identified 66 N-glycan sites per trimeric S-protein unit (24–26). In comparison with other viruses, the S-protein is suggested to have a sparse glycan shield (23). In addition, a site-specific glycan analysis of the S-protein suggests that complex-type (enzyme-modified) N-glycans occupy many of the sites (14 out of 22 sites), whereas high-mannose-type N-glycans commonly dominate in the other viruses (25). The well-known role of a glycan shield is to help virus escaping from host immune systems by hiding active antigen epitopes. In the SARS-CoV-2 S-protein, which has a sparse and complex-type N-glycan rich shield, the glycans likely play not only protective but also passive roles in function. Experimental studies using a combined antigenic screening and cryo-EM structure determination show a possibility to alter the conformational property of S-protein by modifying distinct N-glycans (27,28).

The intrinsic dynamic nature of SARS-CoV-2, as well as the structural and functional roles of the glycosylation, can be overlooked by just structural studies. Molecular dynamics (MD) simulations starting from the atomic structures have opportunities to give complementary information to experimental studies. So far, there exist several reports about modeling of a fully glycosylated full-length SARS-CoV-2 S-protein with palmitoylation at Cys1236 and Cys1241 anchored on a biological membrane (28–31). Although a trimeric structure of SARS-CoV-2 S-protein consisting of S1 and S2 subunits is a large biomolecular complex, the MD-specific supercomputer, Anton2, achieved multiple 10-μs simulations starting from the active and inactive forms (32,33). These simulations provide atomic pictures of a glycan shield hiding the S-protein surface and further predict a passive role of distinct N-glycans to stabilize the active form (30). Thorough analysis of N-glycan heterogeneity at the S-protein surface suggests possible roles of the glycans in modulating the immune response to SARS-CoV-2 virus (28,31). Large-scale simulations of four fully glycosylated full-length S-proteins anchored on a lipid bilayer were carried out to map SARS-CoV-2 epitopes not shielded by N-glycans (15,34). MD simulations also provide structural and energetic details of the interaction between S-protein and human ACE2 receptor, suggesting that balanced hydrogen bonding and hydrophobic contacts make SARS-CoV-2 a stronger binder to human ACE2 than SARS-CoV (35–39).

In this study, we also performed all-atom MD simulations starting from the inactive down and active up forms of a fully glycosylated SARS-CoV-2 S-protein in solution as well as targeted MD (TMD) simulations linking two forms. Our focus is to elucidate key interdomain interactions that regulate conformational stability of each forms by taking into accounts of their dynamic structures. We also ask how the key interactions changes in the conformational transitions between the two forms. Although similar questions were already raised by the previous studies, no systematic analysis has been made using a fully glycosylated SARS-CoV-2 S-protein, to our best knowledge. To answer these questions, atomic interactions involving N-glycans are essential, so we carefully analyze atomic-level interdomain interactions in our simulation trajectories, including the effect of glycosylation. This study will add further mechanistic insight into the highly dynamic nature of S-protein and can help in designing vaccines and antiviral drugs on COVID-19.

METHODS

Atomic structures of SARS-CoV-2 S-protein

Each monomer of a trimeric SARS-CoV-2 S-protein consists of two subunits, the receptor-binding (S1) and the fusion-mediating (S2) subunits (Fig. 1 *A*). The S1 subunit involves the RBD as well as the N-terminal domain (NTD), and several functional regions (upstream helices (UH), fusion peptide (FP), heptad repeats (HR1 and HR2), central helix (CH), and transmembrane domain (TM)) reside in S2 subunit. The RBD binds to the ACE2 receptor to initiate the virus attachment to a host-cell surface, which is followed by the virus-host cell membrane fusion mediated by S1 and S2 (upstream of FP) cleavage and large conformational changes in the S2 subunit. Cryo-EM studies reported two possible conformations of

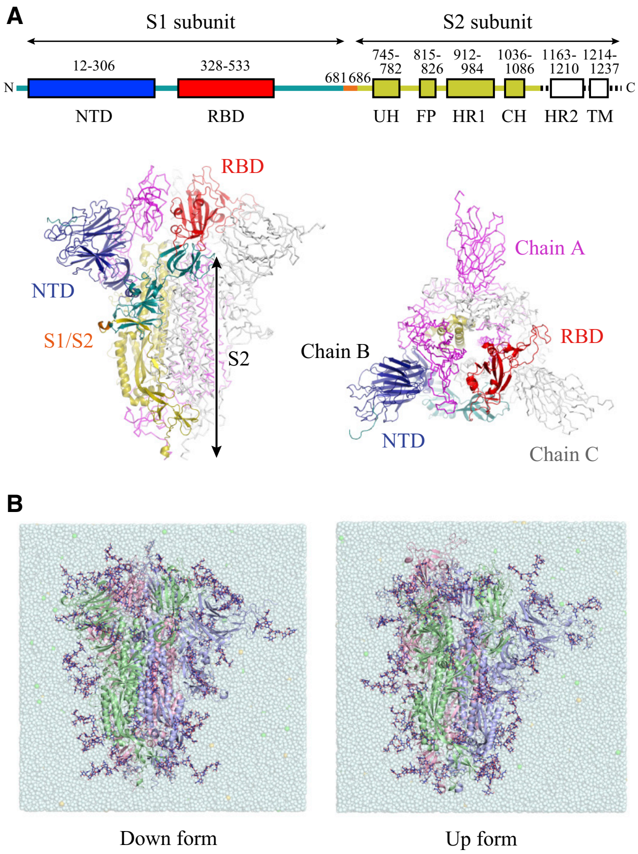

FIGURE 1 The architecture and simulation models of SARS-CoV-2 S-protein. (*A*) The domain architecture of each monomer of a trimeric S-protein is shown (*top*). Here, the chains A and C are represented in magenta and gray, respectively. NTD, RBD, the rest of S1 subunit, and S2 except for HR2 and transmembrane domain in the chain B are highlighted in blue, red, green, and yellow (*bottom*). (*B*) Solvated simulation models of a fully glycosylated S-protein in down (*left*) and up (*right*) forms are shown. The chains A, B, and C are shown in red, green, and blue, respectively. The glycans are shown in blue stick representations. Throughout this study, RBD in chain A can take the up form in MD simulations and undergo the transition between down and up forms in TMD simulations. To see this figure in color, go online.

RBDs, namely down (PDB: 6VXX) (9) and up (PDB: 6VSB) (16). Our simulations started from the solvated models of either the down or the up form, which are available in the CHARMM-GUI COVID-19 Archive (http://www.charmm-gui.org/docs/archive/covid19) (Fig. 1 *B*; (29)). The detailed modeling procedure can be found in the original work by Woo and coworkers (29). In brief, the models include the N-terminus to the residue right before the HR linker in the S2 subunit (residues 1–1146). The missing residues in the original PDB structures were modeled by a template-based modeling using GalaxyTBM (PDB: 6M17 as a template) for the missing loops in the RBD region (residues 336–518) and using the fragment assembly and loop closure program for other missing loops (residues 67–78, 143–155, 177–186, 247–260, and 673–686) (40,41). The long N-terminal region (residues 1–26) and the rest of the missing parts were remodeled based on the electron density map by the ISOLDE software package (42). The models introduce 15 disulfide bonds (10 and 5 in the S1 and S2 subunits, respectively) involving Cys480-Cys488 and Cys840-Cys851 in the missing loop region, whose existence was suggested during the fit to the density map. All histidine residues were modeled as singly protonated on ND1 (HSD). Other titratable residues were modeled as a standard state. These models contain 19 N-glycans and 1 O-glycan attached per monomer, according to the recent mass spectrometry data (Fig. S1; (25,43)). The down form contains 762,293 atoms with box lengths of ∼196 Å, whereas the up form contains 773,227 atoms with box lengths of ∼197 Å after equilibration. Before starting simulations, we replaced counterions K^+ in the original model with Na^+, considering the ion concentration in the extracellular region.

All-atom MD simulations

After 5000-step minimization and equilibration (in total, 652 ps), we performed MD simulations with the isothermal condition at 310.15 K using Bussi's stochastic velocity rescaling thermostat (44). Energy and force are evaluated based on the CHARMM force fields (45–48). The smooth particle mesh Ewald (PME) scheme (49) was used for electrostatic interaction with (128, 128, 128) grids and the PME spline order of 6. We employed a multiple time-step integration using r-RESPA (50) with a time step of 2.5 fs, in which the PME reciprocal-space interaction was evaluated every other steps. The real-space nonbonded interaction was obtained with the cutoff value of 12.0 Å. The van der Waals interaction was evaluated using the force-based switch function acting on the range from 10.0 to 12.0 Å (51). All MD simulations were performed by the new version of GENESIS MD software (52–54). The performance of the S-protein simulations was 68 and 12 ns/day using 128 nodes in Fugaku and 32 nodes in Oakforest-PACS, respectively. For each form, we performed two independent MD simulations for 1 μs and 200 ns by changing the random numbers for initial velocity. Hereafter, we denote them as MD1_Up (1-μs MD from up), MD2_Up (200-ns MD from up), MD1_Down (1-μs MD from down), and MD2_Down (200-ns from down).

TMD simulations

We also performed TMD simulations (55), which give one of the available transition pathways between the starting and target structures by applying external forces. The external force is given to hold the root mean-square deviation (RMSD) from the target structure to a certain value at each time step in the MD simulation. The constraint is calculated using the mass-weighted RMSD of all the heavy atoms of three protein chains except for N-glycans. Therefore, this TMD is useful to examine whether the glycan-protein interactions observed in the conventional MD simulations depend on the initial computational models of N-glycans predicted by CHARMM-GUI tools. The target RMSD value decreases linearly with each time step, and it becomes sufficiently small in the final step. In this simulation, we set 0.5 Å as the final value to avoid too-large constraint forces required for small target RMSD values. TMD simulations were conducted for the transitions from down to up as well as those from up to down. In this study, the starting structure was taken from the snapshot at 50 ns of MD2_Up or MD2_Down, and the target structure was the initial one of MD2_Down or MD2_Up. By changing random seeds for thermostat, we executed three independent TMD simulations with two target directions. 20-, 20-, and 50-ns TMD simulations from down to up forms are denoted as TMD1_ToUp, TMD2_ToUp, and TMD3_ToUp, respectively. 20-, 20-, and 50-ns TMDs from up to down are referred to as TMD1_ToDown, TMD2_ToDown, and TMD3_ToDown, respectively. The simulation methods such as force fields, nonbonded parameters, integrators, and so on are common to those in the conventional MD simulations.

Analysis of interdomain contacts and electrostatic potential

Contact pairs between amino acids or amino acid-glycan in the MD trajectories were analyzed based on the minimal distance between residues including hydrogen. The contact was defined if the minimal distance is

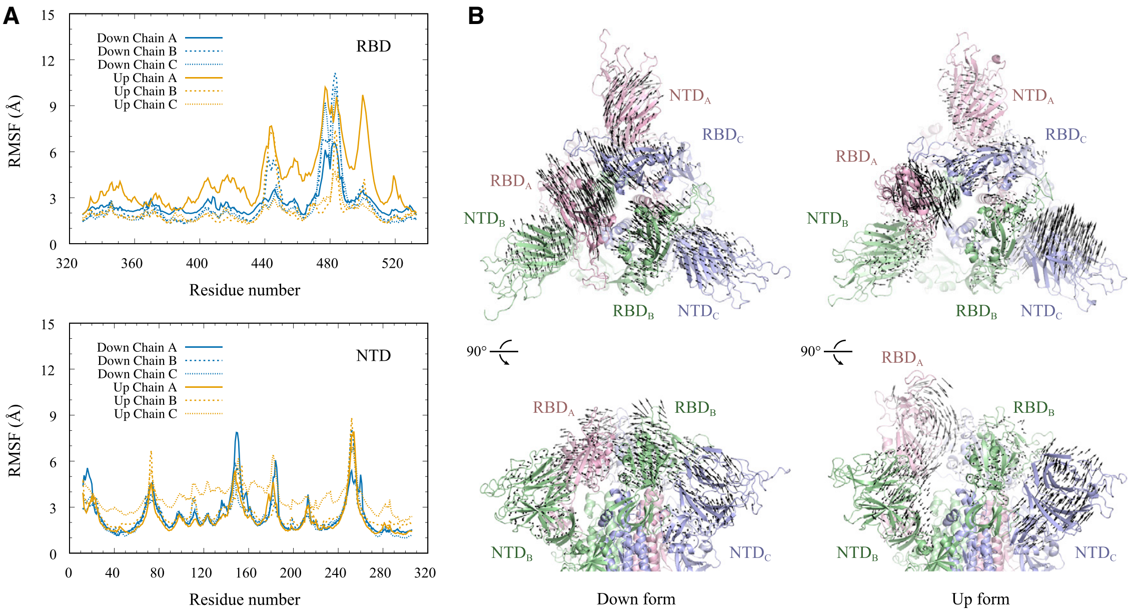

FIGURE 2 (*A*) RMSFs of the Cα atoms in residues belong to RBDs (*top*) and NTDs (*bottom*) in the last 500 ns of MD1_Down and MD1_Up. (*B*) The lowest mode in PCA of the simulation trajectories of down (*left*) and up (*right*) forms is shown. For clarity, the vectors are magnified 100 times. Both top and side views are shown in top and bottom figures, respectively. To obtain the RMSF and PCA, the S2 subunit of simulation snapshots was superimposed to that in the down form of S-protein. To see this figure in color, go online.

less than 2.5 Å. The electrostatic potential of the domain surface was calculated for the last snapshot of the MD simulation using the APBS (adaptive Poisson-Boltzmann solver) tool implemented in PyMOL (56,57). The linear Poisson-Boltzmann equation was solved at 150 mM ionic strength in monovalent salt with a solvent dielectric of 78.0 and a solute dielectric of 2.0. The CHARMM C36m force-field parameters (atomic charge and radius) were applied to the amino acids and amino acid-glycans.

RESULTS AND DISCUSSION

Conformational fluctuations of SARS-CoV-2 S-protein in solution

In the four MD simulations, the overall structures of SARS-CoV-2 S-protein were stable. No transitions between down and up forms were observed for the short timescales. Three RBDs were generally stable as rigid domains in the down-form MD simulations (MD1_Down (Fig. S2 *A*) and MD2_Down (Fig. S3 *A*)). In MD1_Down, RBD$_A$ moved slightly toward the up form (Fig. S2 *A*). Larger RMSDs of both RBDs are observed in MD1_Up (Fig. S2 *B*) and MD2_Up (Fig. S3 *B*). In contrast, NTDs in the down and up forms are almost equally rigid (Figs. S2 and S3). In MD1_Up, rigid domain movements of NTD$_B$ and NTD$_C$ were observed around 400 and 650 ns, respectively (RMSD $\sim$10 Å). Therefore, we mainly focus on the last 500 ns of MD1_Up and MD1_Down, which are considered to be fully equilibrated.

Figs. 2 and S4 show the root mean-square fluctuations (RMSFs) and the lowest mode vectors in principal component analysis (PCA) of three RBDs and NTDs in MD1_Down/Up and MD2_Down/Up, respectively. In these analyses, the S2 subunit of simulation snapshots was superimposed to that of the down form of the cryo-EM structure (PDB: 6VXX). The flexible loop regions that were not determined with cryo-EM analysis were omitted in these analyses. The RMSF analysis suggests the existence of large conformational fluctuations of the loop region around the C480 residue in both down and up forms. The RMSFs of three RBDs are almost comparable in MD1_Down, whereas RMSF of RBD$_A$ is larger than RBD$_B$ and RBD$_C$ in MD1_UP (Fig. 2). The RMSFs of three NTDs in the down and up forms are almost comparable, except for NTD$_C$ in Up form. This is mainly due to a rigid domain movement observed around 650 ns of MD1_Up. The RMSFs of RBDs in MD2_Up are larger than those in MD2_Down (Fig. S4). In three RBDs of the up form, the RMSFs of RBD$_A$ are the largest. Because RBD$_A$ takes the up conformation, it reduces interdomain contacts with the other domains significantly. The RMSFs of NTDs in the up form are also greater than those in the down form, and the order of magnitude is NTD$_B$ $\approx$ NTD$_C$ $>$ NTD$_A$. When RBD$_A$ takes the up form, NTD$_B$ and NTD$_C$ become more mobile.

PCA on MD1_Down/Up suggests collective motions of three RBDs and NTDs in S-protein both in the down and up forms. In particular, RBD$_A$-NTD$_B$, RBD$_B$-NTD$_C$, and RBD$_C$-NTD$_A$ in down move in the same directions (Fig. 2 *B*, *top view*) in the lowest PC mode, which contributes $\sim$30% of total conformational fluctuations. From the side view, the whole RBDs and NTDs in down seem to undergo

swinging motions along the lowest PC mode. In contrast, the domain motions in up seem to be less cooperative. RBD_A moves toward the cavity between NTD_B and RBD_B. RBD_C becomes less mobile because of interactions with RBD_A, which might make NTD_A less mobile. The contribution of the lowest PC mode in MD1_Up to the total conformational fluctuations is ~35%. On the other hand, in the MD2 simulation, NTD_B moves toward the cavity and RBD_A and RBD_C move together because of their direct interactions (Fig. S4). As a result, the fluctuations of NTD_A become smaller. This PCA suggests that the change of RBD_A position increases the mobility of NTD_B and RBD_B, which are adjacent to RBD_A. The increased mobility of another RBD may induce the structure toward a two-RBDs-up conformation, which was recently determined using cryo-EM (12).

Interdomain residue-residue and residue-glycan interactions in MD simulations

In Fig. 3, we show the probability of forming the interdomain contacts and hydrogen bonds in MD1_Down and MD1_Up. Only the results of residue pairs that have more than 55% (Fig. 3, *A*, *B*, and *D*) or 20% (Fig. 3 *C*) of the averaged contacts in either MD1_Down or MD1_Up are shown. In MD1_Down, some residue-residue and residue-glycan contacts exist between different RBDs (Fig. 3 *A*). A complex-type N-glycan attached at N343 forms contacts and hydrogen bonds with Y489 and Q493, suggesting a major role in the conformational stability of RBDs (Fig. 4 *A*, *top*). The patterns of the interdomain contacts and hydrogen bonds change drastically from down to up. In the up form, no interactions between RBD_A and RBD_B are found, whereas the contacts and hydrogen bonds between RBD_A and RBD_C are emphasized; S477-T385, Q493-C379, Y489-T385, and Q493-K378 pairs provide essential interdomain contacts. The first two result from hydrogen-bond interactions, suggesting their strong influences on the tight interaction between RBD_A and RBD_C. The rest of interactions provide hydrophobic contacts to support the up conformation. Interestingly, Y489 and Q493 switched the interaction pairs before and after the conformational transition (Fig. 4 *A*, *bottom*).

In Fig. 3 *B*, we observed many interdomain contacts and hydrogen bonds between residue pairs and residue-glycan pairs between RBDs and NTDs. In particular, E465-N234 and E516-Y200 pairs include side-chain contacts as well as hydrogen bonds. Some of N-glycans interact with multiple side chains, likely adding their hydrophobic interactions to the stabilization. In particular, a high-mannose-type N-glycan at N234 strongly interacts with S459 and K462, whereas the glycan at N165 seems to loosely contact with various residues (Fig. 4 *B*, *top*). In the up form, most of the contacts and hydrogen bonds in the RBD_A-NTD interface are lost. Loose contacts between N165 and RBD_A and also hydrogen bonds between H519 and N234 play an important role (Fig. 4 *B*, *bottom*). The high-mannose-type N-glycan at N234 intrudes a cavity between NTD_B and RBD_B and interacts with D985 and R983 in $S2_B$, and also T415 in RBD_C (Fig. 3 *C*). Interestingly, most of the contacts and hydrogen bonds between RBD_B and NTD_C, as well as those between RBD_C-NTD_A, are lost in the up form. This suggests the increased flexibility of RBD_B, RBD_C, and three NTDs after taking the up form of RBD_A, which is consistent with our observations of RMSFs (Fig. 2 *A*). Movement of the N-glycans will be further discussed in the analysis of TMD.

The down form contains many contacts among three NTDs and S2 subunit as shown in Fig. 3 *D*. The contacts mainly consist of residue-residue interactions. In particular, hydrogen bonds between S383 and R983 or between S383 and D985 seem to be dominant interactions (Fig. 4 *C*, *top*). Compared with the interactions between RBDs (Fig. 3 *A*) or between RBDs and NTDs (Fig. 3 *B*), electrostatic interactions involving Glu, Asp, and Arg seem to be important in the domain interfaces between RBDs and S2 subunit. All the contacts and hydrogen bonds between RBD_A and S2 disappear in the up form (Fig. 4 *C*, *bottom*), whereas some remain between RBD_B and RBD_C and the S2 subunit. Alternatively, R983 and D985 in S2 interact with the glycan at N234 (Fig. 4 *B*, *bottom*). As we see in Fig. 3 *B*, RBD_A-NTD_B interactions are mainly stabilized through the glycans at N165 and N234. In addition, the intruded N234 mediates the interaction between RBD_A and the S2 subunit. These interactions likely contribute to the stabilization of the up form. Detailed interactions between amino acid residues and glycosylated amino-acid residues N165, N234, and N343 in MD1_Down/Up are illustrated in Fig. S5.

The residue-level interdomain contacts and hydrogen bonds are influenced by subtle changes of domain motions in MD simulations. To examine the statistical significance in the analysis, we examined independent trajectories from MD2_Down/Up (Fig. S6). Because of the conformational stability of the three RBDs in the down form, the results of interdomain contacts and hydrogen bonds in MD2_Down are similar to those in MD1_Down. In contrast, RBD_A in the trajectory of MD1_Down shifted slightly outwards, and therefore, novel residue pairs in RBD-RBD and RBD-NTD interfaces are shown in Fig. 3. The residue pairs in RBD-S2 interface are marginally affected, suggesting their strong interactions in the SARS-CoV-2 S-protein. In Figs. S7 and S8, the time series of interdomain contacts in MD1_Down/Up and MD2_Down/Up are shown. We could see that most interaction pairs shown in Fig. 3 are stable over the last 500 ns, demonstrating a good convergence of our simulations.

How do interdomain interactions change in transitions between the down and up forms?

Here, we examine TMD simulation trajectories to answer how the detected interdomain interactions change from

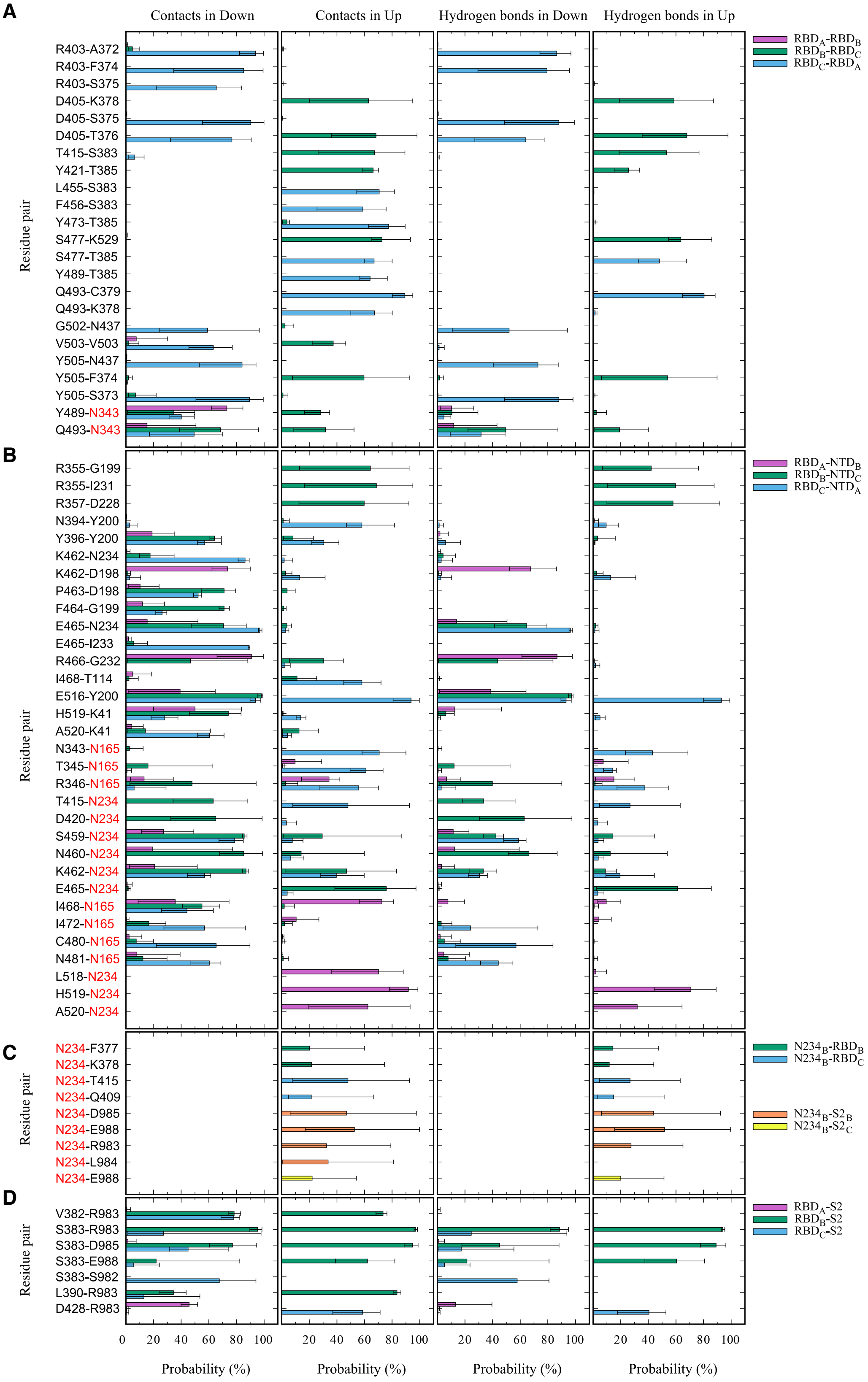

FIGURE 3 The interdomain contacts and hydrogen bonds in MD1_Down and MD1_Up. (*A*) The interactions between different RBDs, RBD_A-RBD_B (*purple*), RBD_B-RBD_C (*green*), and RBD_C-RBD_A (*light blue*); (*B*) those between RBDs and NTDs, RBD_A-NTD_B (*purple*), RBD_B-NTD_C (*green*), and RBD_C-NTD_A (*light blue*); (*C*) those between N234 and RBDs/S2, $N234_B$-RBD_B (*green*), $N234_B$-RBD_C (*light blue*), $N234_B$-$S2_B$ (*orange*), and $N234_B$-$S2_C$ (*yellow*);

(legend continued on next page)

down to up or from up to down transitions. The analysis is necessary to understand the interactions related to the N-glycans because the previous analysis depends on the starting structures of N-glycans in MD simulations. In TMD simulations starting from down to up or vice versa, we added restraints only to the protein heavy atoms, allowing the N-glycans to move freely (Fig. S9). Fig. 5 shows the minimal distances of the interdomain amino acid residue and residue-glycan pairs in TMD3_ToUp (see Fig. S10 for all trajectories). The two amino-acid residue pairs, namely Y489/Q493 in RBD_C and T385/K378 in RBD_A, are broken at the domain interface (Fig. 5, *top left*). Similarly, two pairs between Y489/Q493 in RBD_C and the complex-type N-glycan at N343 in RBD_A break in the middle of the TMD simulation from down to up (Fig. 5, *top left*). Instead, Y489 and Q493 in RBD_C make new contacts with T385 and K378 in RBD_A, respectively, showing the switching of the interaction pairs. Interactions between amino acid residues in RBD_A and a complex-type N-glycan at N165 in NTD_B are basically stable (Fig. 5, *top right*), and some interactions can be transiently formed (e.g., N481-N165). These results suggest that N165 can follow RBD_A during the transition between down and up.

In the RBD-NTD-S2 interface, E516 and S383 in RBD_A lose their interactions with Y200 in NTD_B and R983 in $S2_B$, respectively (Fig. 5, *bottom left*). Similarly, the high-mannose-type N-glycan at N234 in NTD_B loses the interactions with K462 in RBD_A, but it makes a new contact with H519 in RBD_A after the transition to the up form. In addition, the glycan at N234 can form contacts with E988 in both $S2_B$ and $S2_C$, which are located at the top of S2 (Fig. 5, *bottom right*). The glycan at N234 intrudes into the void space between RBD_A, NTD_B, and S2 because of strong electrostatic interactions between the glycan and amino acid residues. The change in RBD_A position during the transition likely weakens the interdomain interaction. The high-mannose-type N-glycan at N234 in NTD_B provides sufficient interdomain contacts between RBD_A, RBD_B, and S2, which seem to be important for the stabilization of the up form of the S-protein. In general, the TMD simulation results (Figs. 5 and S10) are consistent with the interdomain contacts and hydrogen bonds observed in canonical MD simulations (Figs. 3 and S5).

Fig. 6 shows three snapshots of TMD3_ToUp obtained at 0, 25, and 32 ns, whose RMSDs with respect to the up form are 6.8, 3.1, and 2.0 Å, respectively. The glycan at N165 in NTD_B moves upward, following the motion of RBD_A, and fills the cavity between RBD_A and NTD_B. The N-glycan 343 in RBD_A loses the interactions with RBD_C because of the movement of RBD_A. The N-glycan at N234, facing outwards in the down form, fills a cavity formed by the motion of RBD_A. Finally, it reaches the top of the S2 domain. The motions and interactions with the N-glycans suggest that upon the large positional changes of RBD_A from down to up, the N-glycans show significant flexibility in their conformation and change their interaction partners.

Role of electrostatic repulsions between three RBDs in the down form

The analysis of interdomain contacts and hydrogen bonds suggests the importance of charged amino acid residues and their interactions. Fig. 7 shows the surface charge distributions of RBDs at 500 ns of the MD1_Down simulation using the APBS tool in PyMOL software (56,57). In the top view of RBDs (Fig. 7 *A*), we found that the surface of the central cavity formed by three RBDs is positively charged, which may present repulsive forces. The positive charges mainly result from the side chain of K417, R408, and K378, as we see in the side view (Fig. 7 *B*). There exist negatively charged regions near K378; they result from the exposed backbone carbonyl groups in F374 as well as S374 and do not interact with the other RBD surfaces. D405 is close to R408, but the side chain of D405 is not exposed to the RBD interface.

In the up form, K378 in an RBD can interact with Q493 in another nearby RBD (Figs. 3 and 4). The loop structure including Q493 is flexible (Fig. 2). This loop also includes the negatively charged residue E484. Interestingly, E484 in RBD_A frequently formed a hydrogen bond with K378 at the beginning of MD1_Up (the averaged frequency of the hydrogen-bond formation is ~50% in 0–300 ns) and in the middle of TMD simulations. The electrostatic interaction between this flexible loop and positively charged surface of RBD may act as an attractive force toward the up form.

Molecular interactions regulating conformational stability and transitions of SARS-CoV-2 S-protein

In this study, we performed four all-atom MD simulations of SARS-CoV-2 S-protein in solution: two simulations starting from the down form (1 μs and 200 ns) and two from the up form (1 μs and 200 ns). Based on the simulation trajectories, we detect important interdomain interactions as shown in Figs. 3 and S4. Although some of them show different

and (*D*) those between RBDs and S2, RBD_A-S2 (*purple*), RBD_B-S2 (*green*), and RBD_C-S2 (*light blue*), are shown. Red characters mean the glycosylated amino acid residues. In the analysis, a contact is defined when the minimal distance between two residues is shorter than 2.5 Å. A hydrogen bond is decided if the D…A (donor…acceptor) distance is shorter than 3.4 Å, the D-H…A angle is smaller than 120°, and the H-D…A angle is greater than 30°. The last 500-ns trajectory is divided into five 100-ns trajectories, and the average numbers of contacts and hydrogen bonds are shown as bars. The maximal and minimal numbers are shown in error bars. Only the residue pairs that have more than 55% (for *A*, *B*, and *D*) or 20% (for *C*) of contacts in either MD1_Down or MD1_Up are shown. To see this figure in color, go online.

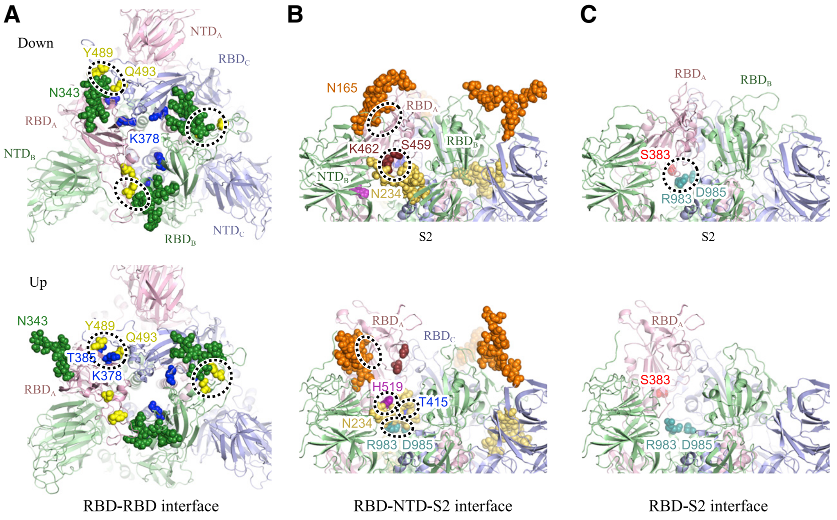

FIGURE 4 Key interdomain interactions at the final snapshots of MD1_Down (*top*) and MD1_Up (*bottom*). (*A*) The RBD-RBD interface, (*B*) the RBD-NTD-S2 interface, and (*C*) the RBD-S2 interface are shown. Dotted circles show strong interactions to stabilize the position of RBDs in each form. Key residues and N-glycans are shown in sphere representations. To see this figure in color, go online.

frequencies of the formation of interdomain contacts and hydrogen bonds, we observed common key features in two independent simulation trajectories in both the down and up forms. The interaction involving the N-glycan is also investigated, and the functional roles of distinct glycans at N165, N234, and N343 are elucidated. However, the glycan interactions can be influenced strongly by the starting structures of MD simulations. The glycan conformations in the structures were added computationally by CHARMM-GUI tools, likely affecting the analysis result. To be independent from possible computational artifacts, we conducted six TMD simulations: three for the transitions from down to up (20, 20, and 50 ns) and three for those from up to down (20, 20, and 50 ns). In the TMD simulations, we did not add restraining forces to the N-glycans in S-protein so the N-glycans can freely fluctuate during the conformational changes toward the up or down form. In the analysis of time courses of the minimal distances, we obtained the consistent results of interdomain interactions with those observed in the canonical MD simulation trajectories. We therefore consider that this analysis of contacts and hydrogen bonds is meaningful in cases of the amino-acid residue pairs as well as N-glycan/amino-acid residue pairs. The key interdomain interactions that stabilize either down or up forms are summarized in Fig. 8. We call this conformational transition mediated by glycans the "glycan-locking mechanism," by which the three major glycans N165, N234, and N343 regulate the movement of RBDs.

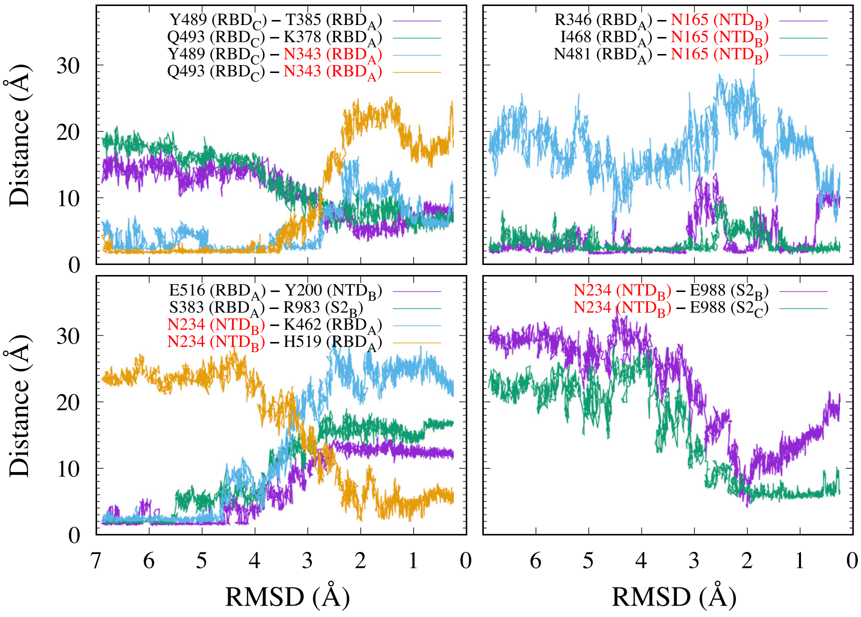

FIGURE 5 The minimal distances of the interdomain amino acid residue and residue-glycan pairs in the TMD2_ToUp simulation. Red characters mean the glycosylated amino acid residues. RMSD in TMD simulations is measured using the cryo-EM structure in the up form as a reference. To see this figure in color, go online.

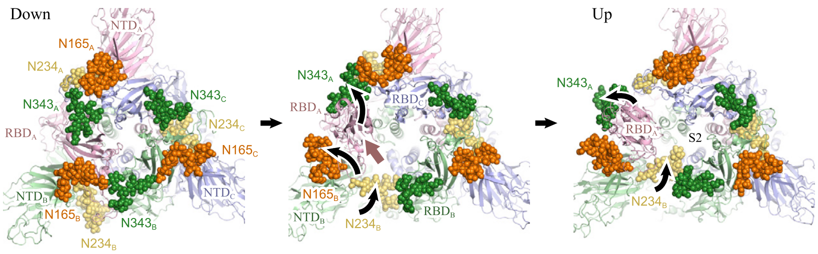

FIGURE 6 Key interdomain interactions observed at the initial (0 ns), 10 ns, and the final (20 ns) of TMD2_ToUp. The figures show a side view of the RBD-NTD interface. Key residues and N-glycans are shown in sphere representations. To see this figure in color, go online.

Casalino et al. carried out all-atom MD simulations of fully glycosylated full-length SARS-CoV-2 S-protein and discussed the role of a glycan shield (30). They compared accessible surface areas of S-protein with and without glycosylation in their multiple-microsecond MD simulations in both the down and up forms. They also discussed the role of N-glycans at N165 and N234 to stabilize the up form and performed additional MD simulations by mutating N165 and N234 to alanine to conform their functional roles. The simulations showed large destabilization of the RBDs in the mutant, confirming the glycans' functions. Our results on the role of N165 and N234 are fully consistent with their finding and add further details on their functional motion. The electrostatic repulsion in the central cavity between three RBDs in the down form is likely to be overlooked in the previous studies. Because N-glycans increase the conformational stability of both the up and down forms in different manners, there must be destabilization factors or driving forces on the conformational changes in the S-protein. We consider that the electrostatic repulsion in the cavity at trimeric center is one such device in the S-protein. We note that there is no N-glycan in the region, so this repulsion is independent from the glycosylation of S-protein.

We performed, in total, 1.2-μs canonical MD simulations of a fully glycosylated SARS-CoV-2 S-protein in solution. The simulation lengths are a bit shorter than other computational studies including multiple-microsecond MD simulations. However, none of them succeeded in simulating the spontaneous conformational changes between up and down in multiple-microsecond MD, suggesting that much longer timescales are required in the conformational changes. In this sense, if we focus on the elucidation of the interdomain interactions regulating the conformational stability of the up and down forms of the S-protein, our simulations seem to be enough, and the results are meaningful. Because N-glycans are very flexible, we might need longer simulations to examine the interactions between N-glycans and amino acids in different domains. Therefore, we performed multiple TMD simulations for investigating such interactions, allowing N-glycans to move freely in TMD simulations. Fortunately, the TMD results agree with the contact and hydrogen-bond analysis of canonical MD simulation trajectories.

Finally, we discuss the conformational changes of the S-protein between the down and up forms. We performed six TMD simulations between the two states and observed the structural changes between them. In TMD simulations, we add the RMSD restraint forces to protein heavy atoms and force the protein toward the target structure. As shown in Fig. S9, the conformational change happened almost linearly without the effect of thermal fluctuations. It was also criticized many times that the pathways obtained in TMD may not be realistic compared to other sampling schemes. In this work, we do not aim to discuss the intermediate structures between the up and down forms of the SARS-CoV-2 S-protein. To discuss the intermediate structures, we suggest performing MD simulations based on enhanced conformational sampling methods, such as replica-exchange schemes (58). Considering the large system size of a fully glycosylated SARS-CoV-2 S-protein in explicit solution, gREST (59) or GaMD (60–62) seems to be a good scheme to be applied rather than the original T-REMD (63). The gREST simulations of the S-protein in solution are now underway in our group using Fugaku supercomputer resources. The results on the spontaneous conformational changes of the S-protein and the intermediate structures between the down and up forms will be discussed elsewhere.

CONCLUSIONS

Based on the fully glycosylated SARS-CoV-2 spike protein structural models, we performed all-atom MD simulations of the up and down forms and target MD simulations between them. To understand key interdomain interactions in each form, we analyzed the simulation trajectories in terms of atomic contacts and hydrogen bonds between different RBDs, between RBDs and NTDs, and between RBDs and the S2 subunit. The down and up forms of the S-protein are stabilized via different residue-residue and/or residue-glycan interactions, as summarized in Fig. 8. In addition to the stabilization factors including the hydrophobic and hydrogen bonding interactions, repulsive forces are implemented in the interior cavity surface formed by three RBDs. The repulsive electrostatic forces between positively charged side chains in three RBDs are balanced

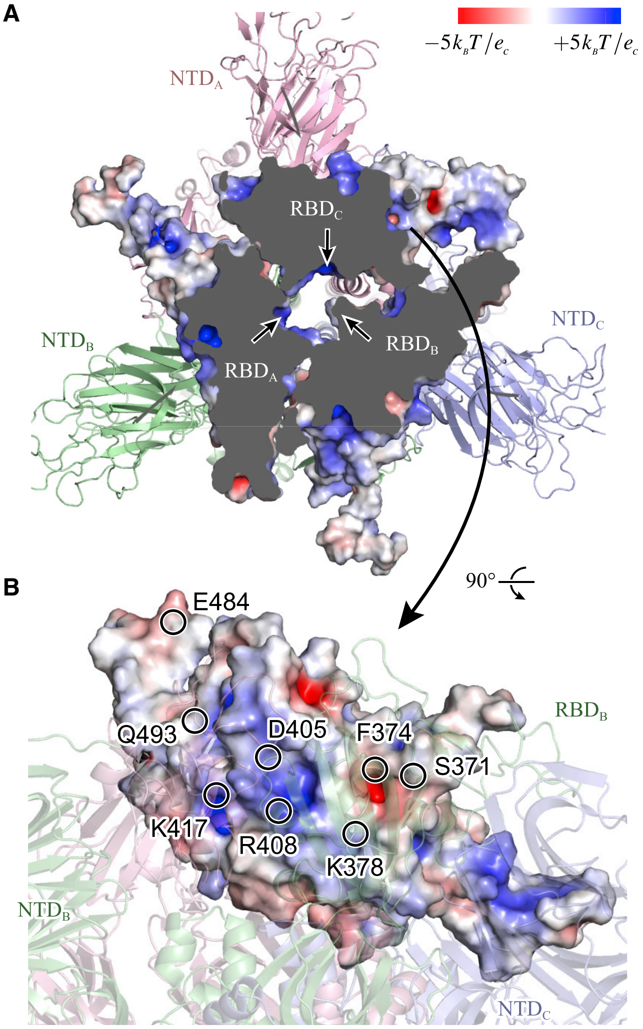

FIGURE 7 Electrostatic potential mapped to RBDs in down form. Three basic amino-acid side chains, K378, R408, and K417, provide positively charged surfaces toward the center of a trimeric form of S-protein. APBS plugin in PyMOL software (56,57) is used to visualize the electrostatic potential. To see this figure in color, go online.

with interdomain hydrogen bonds and van der Waals interactions to stabilize the down form. In the transition from down to up forms, the electrostatic interaction may work as a driving force to break the trimeric symmetry of the S-protein conformation. The up form, which is an asymmetric structure with only one RBD taking the up form, is stabilized via side-chain electrostatic interactions and residue-glycan interactions to fill the cavity between two chains. This study was able to elucidate key atomic interactions to stabilize the down and up forms that were determined by cryo-EM, whereas target MD simulation could predict only one possible pathway between the down and up forms. To find intermediate structures and other candidates of the active conformations, we need to explore the conformational space of SARS-CoV-2 more extensively by using some of the enhanced conformational sampling methods.

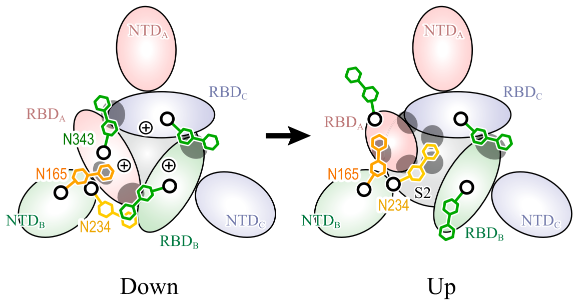

FIGURE 8 "Glycan-locking mechanism" for the conformational transition between down and up forms of SARS-CoV-2 S-protein. Major interdomain interactions observed in all-atom MD and TMD simulations are illustrated as gray ellipsoids. Electrostatic interactions between three RBDs give repulsive forces for driving conformational changes from down to up, whereas interdomain contacts and hydrogen bonds between a trimeric structure stabilize the down form with the help of N-glycans at N343. The up form loses interdomain contacts and hydrogen bonds, in particular between RBD$_B$, RBD$_C$, and S2. However, the N-glycan at N234 supports the interaction between them by intruding into the void space. To see this figure in color, go online.

SUPPORTING MATERIAL

Supporting Material can be found online at https://doi.org/10.1016/j.bpj.2021.01.012.

AUTHOR CONTRIBUTIONS

J.J. and C.K. carried out MD simulations. T.M. and C.K. analyzed simulation data. T.M., J.J., C.K., H.M.D., S.R., and Y.S. wrote the manuscript. T.M. and Y.S. designed research.

ACKNOWLEDGMENTS

This research used computer resources of Fugaku in RIKEN Center for Computational Science (G9330003). The computer resources of Oakforest-PACS were also provided through HPCI System Research project (project ID: hp200153). The research was supported in part by MEXT as "FLAGSHIP 2020 project," "Priority Issue on Post-K computer" (Building Innovative Drug Discovery Infrastructure Through Functional Control of Biomolecular Systems), "Program for Promoting Researches on the Supercomputer Fugaku" (Biomolecular dynamics in a living cell/MD-driven Precision Medicine), and MEXT/KAKENHI (Grant Numbers 19H05645 (to Y.S.), 19K12229 (to S.R.), and 19K06532 (to T.M.)) and by RIKEN Pioneering Research Projects (Dynamic Structural Biology/Glycolipidologue Initiative) (to Y.S.). The results obtained on the evaluation environment in the trial phase do not guarantee the performance, power and other attributes of the supercomputer Fugaku at the start of its operation.

REFERENCES

1. Chan, J. F.-W., S. Yuan, …, K.-Y. Yuen. 2020. A familial cluster of pneumonia associated with the 2019 novel coronavirus indicating person-to-person transmission: a study of a family cluster. *Lancet.* 395:514–523.

2. Wu, F., S. Zhao, …, Y. Z. Zhang. 2020. A new coronavirus associated with human respiratory disease in China. *Nature.* 579:265–269.

3. Zhou, P., X.-L. Yang, …, Z.-L. Shi. 2020. A pneumonia outbreak associated with a new coronavirus of probable bat origin. *Nature*. 579:270–273.

4. Liu, C., Q. Zhou, …, D. Albaiu. 2020. Research and development on therapeutic agents and vaccines for COVID-19 and related human coronavirus diseases. *ACS Cent. Sci.* 6:315–331.

5. Jeon, S., M. Ko, …, S. Kim. 2020. Identification of antiviral drug candidates against SARS-CoV-2 from FDA-approved drugs. *Antimicrob. Agents Chemother.* 64:e00819–e00820.

6. Zhang, Y., and T. G. Kutateladze. 2020. Molecular structure analyses suggest strategies to therapeutically target SARS-CoV-2. *Nat. Commun.* 11:2920.

7. Shang, J., Y. Wan, …, F. Li. 2020. Cell entry mechanisms of SARS-CoV-2. *Proc. Natl. Acad. Sci. USA*. 117:11727–11734.

8. Kirchdoerfer, R. N., C. A. Cottrell, …, A. B. Ward. 2016. Pre-fusion structure of a human coronavirus spike protein. *Nature*. 531:118–121.

9. Walls, A. C., Y. J. Park, …, D. Veesler. 2020. Structure, function, and antigenicity of the SARS-CoV-2 spike glycoprotein. *Cell*. 181:281–292.e6.

10. Hoffmann, M., H. Kleine-Weber, …, S. Pöhlmann. 2020. SARS-CoV-2 cell entry depends on ACE2 and TMPRSS2 and is blocked by a clinically proven protease inhibitor. *Cell*. 181:271–280.e8.

11. Berman, H. M., T. Battistuz, …, C. Zardecki. 2002. The Protein Data Bank. *Acta Crystallogr. D Biol. Crystallogr.* 58:899–907.

12. Ke, Z., J. Oton, …, J. A. G. Briggs. 2020. Structures and distributions of SARS-CoV-2 spike proteins on intact virions. *Nature*. 588:498–502.

13. Henderson, R., R. J. Edwards, …, P. Acharya. 2020. Controlling the SARS-CoV-2 spike glycoprotein conformation. *Nat. Struct. Mol. Biol.* 27:925–933.

14. Cai, Y., J. Zhang, …, B. Chen. 2020. Distinct conformational states of SARS-CoV-2 spike protein. *Science*. 369:1586–1592.

15. Turoňová, B., M. Sikora, …, M. Beck. 2020. In situ structural analysis of SARS-CoV-2 spike reveals flexibility mediated by three hinges. *Science*. 370:203–208.

16. Wrapp, D., N. Wang, …, J. S. McLellan. 2020. Cryo-EM structure of the 2019-nCoV spike in the prefusion conformation. *Science*. 367:1260–1263.

17. Barnes, C. O., A. P. West, Jr., …, P. J. Bjorkman. 2020. Structures of human antibodies bound to SARS-CoV-2 spike reveal common epitopes and recurrent features of antibodies. *Cell*. 182:828–842.e16.

18. Yan, R., Y. Zhang, …, Q. Zhou. 2020. Structural basis for the recognition of SARS-CoV-2 by full-length human ACE2. *Science*. 367:1444–1448.

19. Shang, J., G. Ye, …, F. Li. 2020. Structural basis of receptor recognition by SARS-CoV-2. *Nature*. 581:221–224.

20. Lan, J., J. Ge, …, X. Wang. 2020. Structure of the SARS-CoV-2 spike receptor-binding domain bound to the ACE2 receptor. *Nature*. 581:215–220.

21. Yuan, M., N. C. Wu, …, I. A. Wilson. 2020. A highly conserved cryptic epitope in the receptor binding domains of SARS-CoV-2 and SARS-CoV. *Science*. 368:630–633.

22. Wrapp, D., D. De Vlieger, …, J. S. McLellan; VIB-CMB COVID-19 Response Team. 2020. Structural basis for potent neutralization of betacoronaviruses by single-domain camelid antibodies. *Cell*. 181:1004–1015.e15.

23. Watanabe, Y., T. A. Bowden, …, M. Crispin. 2019. Exploitation of glycosylation in enveloped virus pathobiology. *Biochim. Biophys. Acta, Gen. Subj.* 1863:1480–1497.

24. Watanabe, Y., Z. T. Berndsen, …, M. Crispin. 2020. Vulnerabilities in coronavirus glycan shields despite extensive glycosylation. *Nat. Commun.* 11:2688.

25. Watanabe, Y., J. D. Allen, …, M. Crispin. 2020. Site-specific glycan analysis of the SARS-CoV-2 spike. *Science*. 369:330–333.

26. Botten, J., J. Alexander, …, M. J. Buchmeier. 2006. Identification of protective Lassa virus epitopes that are restricted by HLA-A2. *J. Virol.* 80:8351–8361.

27. Henderson, R., R. J. Edwards, …, P. Acharya. 2020. Glycans on the SARS-CoV-2 spike control the receptor binding domain conformation. *bioRxiv* https://doi.org/10.1101/2020.06.26.173765.

28. Zhao, P., J. L. Praissman, …, L. Wells. 2020. Virus-receptor interactions of glycosylated SARS-CoV-2 spike and human ACE2 receptor. *Cell Host Microbe*. 28:586–601.e6.

29. Woo, H., S.-J. Park, …, W. Im. 2020. Developing a fully glycosylated full-length SARS-CoV-2 spike protein model in a viral membrane. *J. Phys. Chem. B*. 124:7128–7137.

30. Casalino, L., Z. Gaieb, …, R. E. Amaro. 2020. Beyond shielding: the roles of glycans in the SARS-CoV-2 spike protein. *ACS Cent. Sci.* 6:1722–1734.

31. Grant, O. C., D. Montgomery, …, R. J. Woods. 2020. Analysis of the SARS-CoV-2 spike protein glycan shield reveals implications for immune recognition. *Sci. Rep.* 10:14991.

32. D. E. Shaw Research. 2020. Molecular dynamics simulations related to SARS-CoV-2, D. E. Shaw Research Technical Data http://www.deshawresearch.com/resources_sarscov2.html.

33. Shaw, D. E., J. P. Grossman, …, C. Young. 2014. Anton 2: raising the bar for performance and programmability in a special-purpose molecular dynamics supercomputer. *In* SC14: International Conference for High Performance Computing, Networking, Storage and Analysis. IEEE, pp. 41–53.

34. Sikora, M., M. Gecht, …, G. Hummer. 2020. Map of SARS-CoV-2 spike epitopes not shielded by glycans. *bioRxiv* https://doi.org/10.1101/2020.07.03.186825.

35. Wang, Y., M. Liu, and J. Gao. 2020. Enhanced receptor binding of SARS-CoV-2 through networks of hydrogen-bonding and hydrophobic interactions. *Proc. Natl. Acad. Sci. USA*. 117:13967–13974.

36. Brielle, E. S., D. Schneidman-Duhovny, and M. Linial. 2020. The SARS-CoV-2 exerts a distinctive strategy for interacting with the ACE2 human receptor. *Viruses*. 12:497.

37. Corrêa Giron, C., A. Laaksonen, and F. L. Barroso da Silva. 2020. On the interactions of the receptor-binding domain of SARS-CoV-1 and SARS-CoV-2 spike proteins with monoclonal antibodies and the receptor ACE2. *Virus Res.* 285:198021.

38. Dehury, B., V. Raina, …, M. Suar. 2020. Effect of mutation on structure, function and dynamics of receptor binding domain of human SARS-CoV-2 with host cell receptor ACE2: a molecular dynamics simulations study. *J. Biomol. Struct. Dyn*, Published online August 7, 2020.

39. Zou, J., J. Yin, …, P. Zhang. 2020. Computational prediction of mutational effects on SARS-CoV-2 binding by relative free energy calculations. *J. Chem. Inf. Model.* 60:5794–5802.

40. Ko, J., D. Lee, …, C. Seok. 2011. The FALC-Loop web server for protein loop modeling. *Nucleic Acids Res.* 39:W210–W214.

41. Ko, J., H. Park, and C. Seok. 2012. GalaxyTBM: template-based modeling by building a reliable core and refining unreliable local regions. *BMC Bioinformatics*. 13:198.

42. Croll, T. I. 2018. ISOLDE: a physically realistic environment for model building into low-resolution electron-density maps. *Acta Crystallogr. D Struct. Biol.* 74:519–530.

43. Shajahan, A., N. T. Supekar, …, P. Azadi. 2020. Deducing the N- and O-glycosylation profile of the spike protein of novel coronavirus SARS-CoV-2. *Glycobiology*. 30:981–988.

44. Bussi, G., D. Donadio, and M. Parrinello. 2007. Canonical sampling through velocity rescaling. *J. Chem. Phys.* 126:014101.

45. Guvench, O., S. N. Greene, …, A. D. Mackerell, Jr. 2008. Additive empirical force field for hexopyranose monosaccharides. *J. Comput. Chem.* 29:2543–2564.

46. Klauda, J. B., R. M. Venable, …, R. W. Pastor. 2010. Update of the CHARMM all-atom additive force field for lipids: validation on six lipid types. *J. Phys. Chem. B*. 114:7830–7843.

47. Best, R. B., X. Zhu, …, A. D. Mackerell, Jr. 2012. Optimization of the additive CHARMM all-atom protein force field targeting improved sampling of the backbone φ, ψ and side-chain χ(1) and χ(2) dihedral angles. *J. Chem. Theory Comput.* 8:3257–3273.

48. Huang, J., S. Rauscher, …, A. D. MacKerell, Jr. 2017. CHARMM36m: an improved force field for folded and intrinsically disordered proteins. *Nat. Methods.* 14:71–73.

49. Essmann, U., L. Perera, …, L. G. Pedersen. 1995. A smooth particle mesh Ewald method. *J. Chem. Phys.* 103:8577–8593.

50. Tuckerman, M., B. J. Berne, and G. J. Martyna. 1992. Reversible multiple time scale molecular dynamics. *J. Chem. Phys.* 97:1990–2001.

51. Steinbach, P. J., and B. R. Brooks. 1994. New spherical-cutoff methods for long-range forces in macromolecular simulation. *J. Comput. Chem.* 15:667–683.

52. GENESIS-2.0. https://github.com/genesis-release-r-ccs/genesis-2.0.

53. Kobayashi, C., J. Jung, …, Y. Sugita. 2017. GENESIS 1.1: a hybrid-parallel molecular dynamics simulator with enhanced sampling algorithms on multiple computational platforms. *J. Comput. Chem.* 38:2193–2206.

54. Jung, J., T. Mori, …, Y. Sugita. 2015. GENESIS: a hybrid-parallel and multi-scale molecular dynamics simulator with enhanced sampling algorithms for biomolecular and cellular simulations. *Wiley Interdiscip. Rev. Comput. Mol. Sci.* 5:310–323.

55. Schlitter, J., M. Engels, …, A. Wollmer. 1993. Targeted molecular dynamics simulation of conformational change-application to the T ↔ R transition in insulin. *Mol. Simul.* 10:291–308.

56. Baker, N. A., D. Sept, …, J. A. McCammon. 2001. Electrostatics of nanosystems: application to microtubules and the ribosome. *Proc. Natl. Acad. Sci. USA.* 98:10037–10041.

57. Schrödinger, LLC.. 2020. The PyMOL Molecular Graphics System, Version 2.4.

58. Sugita, Y., M. Kamiya, …, S. Re. 2019. Replica-exchange methods for biomolecular simulations. *Methods Mol. Biol.* 2022:155–177.

59. Kamiya, M., and Y. Sugita. 2018. Flexible selection of the solute region in replica exchange with solute tempering: application to protein-folding simulations. *J. Chem. Phys.* 149:072304.

60. Oshima, H., S. Re, and Y. Sugita. 2019. Replica-exchange umbrella sampling combined with Gaussian accelerated molecular dynamics for free-energy calculation of biomolecules. *J. Chem. Theory Comput.* 15:5199–5208.

61. Miao, Y., V. A. Feher, and J. A. McCammon. 2015. Gaussian accelerated molecular dynamics: unconstrained enhanced sampling and free energy calculation. *J. Chem. Theory Comput.* 11:3584–3595.

62. Pang, Y. T., Y. Miao, …, J. A. McCammon. 2017. Gaussian accelerated molecular dynamics in NAMD. *J. Chem. Theory Comput.* 13:9–19.

63. Sugita, Y., and Y. Okamoto. 1999. Replica-exchange molecular dynamics method for protein folding. *Chem. Phys. Lett.* 314:141–151.

Biophysical Journal, Volume *120*

Supplemental information

Elucidation of interactions regulating conformational stability and dynamics of SARS-CoV-2 S-protein

Takaharu Mori, Jaewoon Jung, Chigusa Kobayashi, Hisham M. Dokainish, Suyong Re, and Yuji Sugita

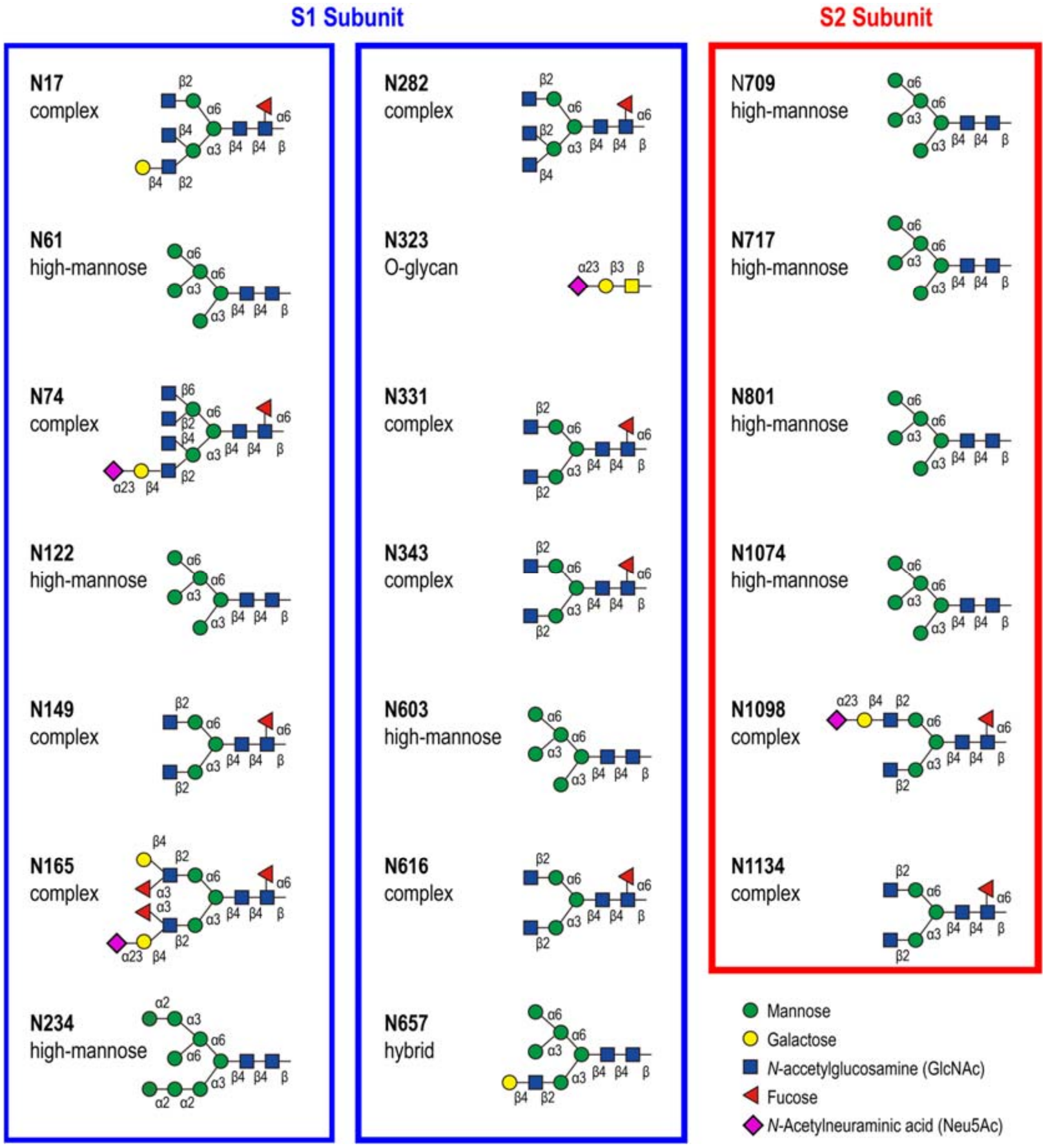

Figure S1. Summary of glycan structures and types attached to the S1 and S2 subunits of the S-protein used in all-the current all-atom MD simulations.

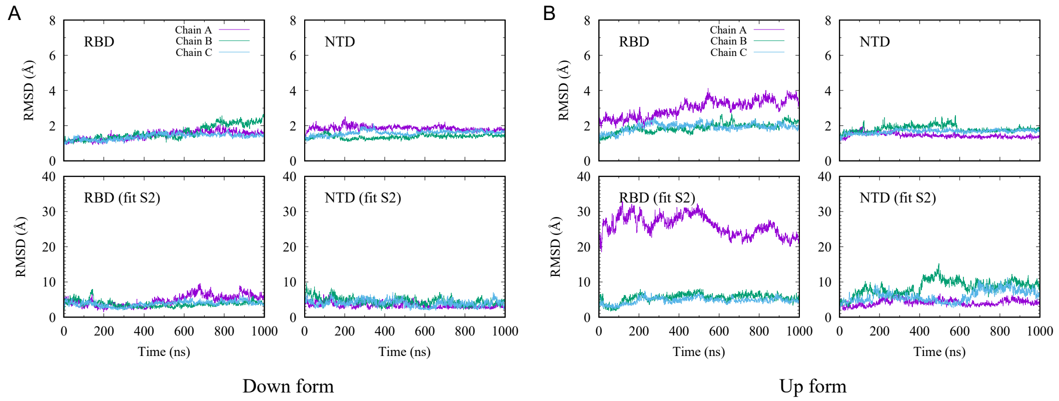

Figure S2. Root-mean-square deviations (RMSDs) of RBD and NTD in S-protein obtained in MD1_Down (A) and MD1_Up (B). In each graph, domains in chains A, B, and C are shown in purple, green, and light blue. In the calculations of RMSD, the superimposed regions in top and bottom are RBD/NTD itself and the S2 subunit in the Down form, respectively.

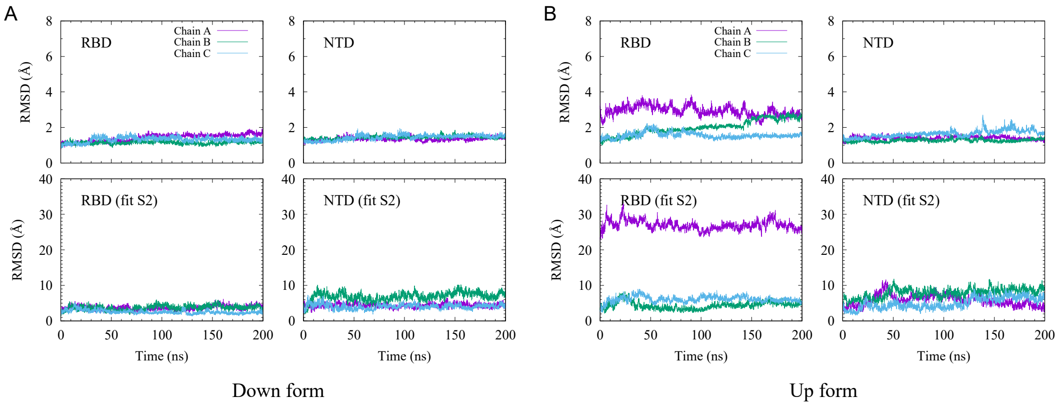

Figure S3. Root-mean-square deviations (RMSDs) of RBD and NTD in S-protein obtained in MD2_Down (A) and MD2_Up (B). In each graph, domains in chains A, B, and C are shown in purple, green, and light blue. In the calculations of RMSD, the superimposed regions in top and bottom are RBD/NTD itself and the S2 subunit in the Down form, respectively.

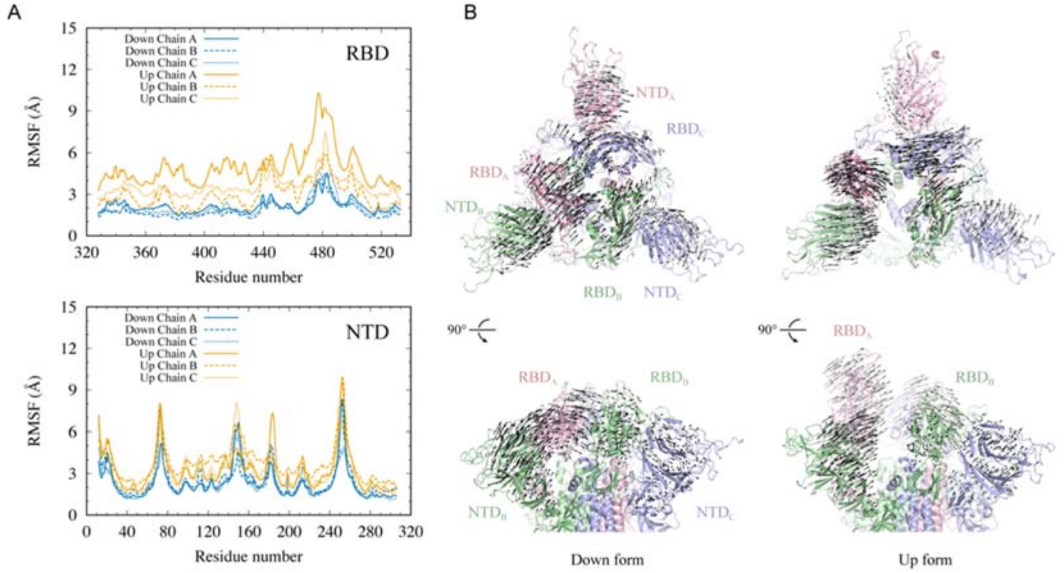

Figure S4. (A) Root-mean-square fluctuations (RMSFs) of the Cα atoms in residues belong to RBD (top) and NTD (bottom) in MD2_Down and MD2_Up. The lowest mode in principal component analysis (PCA) of the simulation trajectories of Down (left) and Up (right) forms. For clarity, the vectors are magnified 100 times. Both top and side views are shown in top and bottom figures, respectively. To obtain the RMSF and PCA, the S2 subunit of simulation snapshots was superimposed to that in Down form of S-protein.

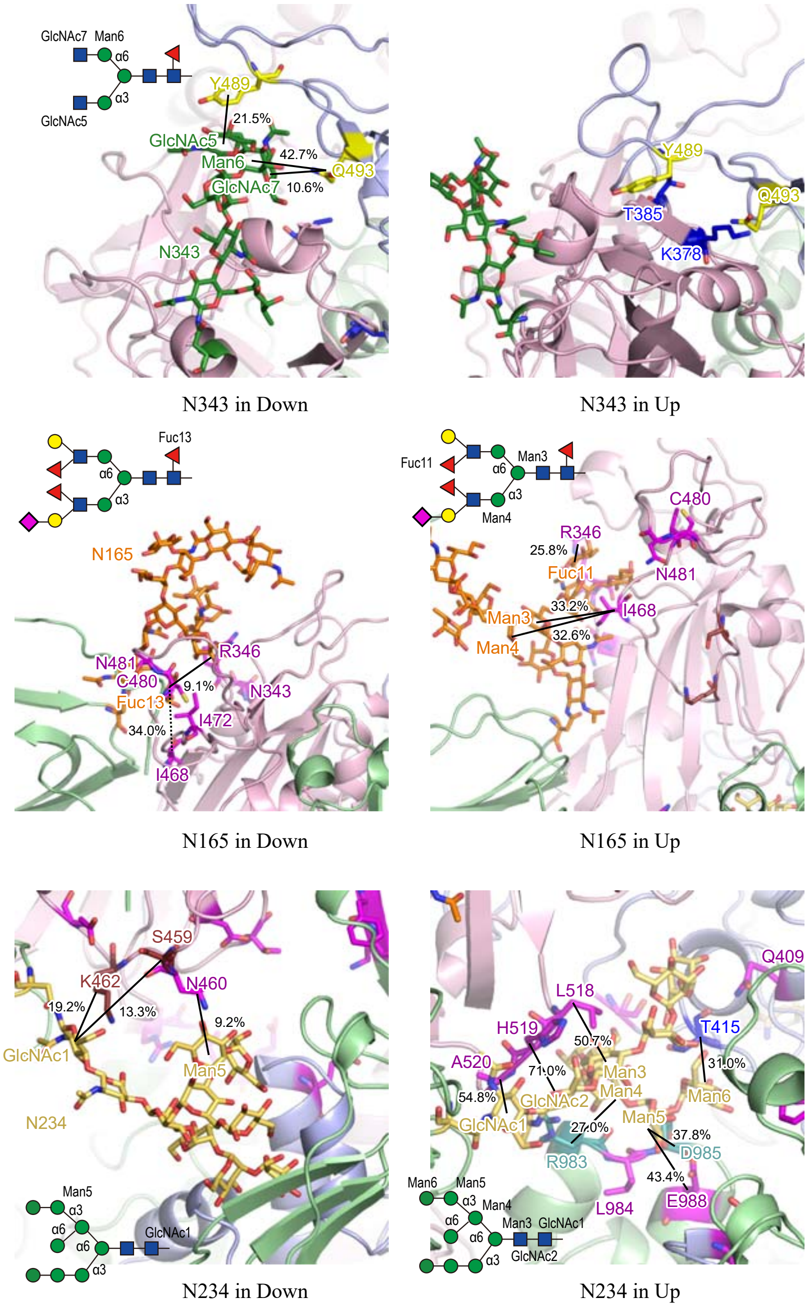

Figure S5. Interactions between amino acids and glycans at N343, N165, and N234. The probabilities of the contact formation in the last 500 ns of MD1_Down/Up are shown.

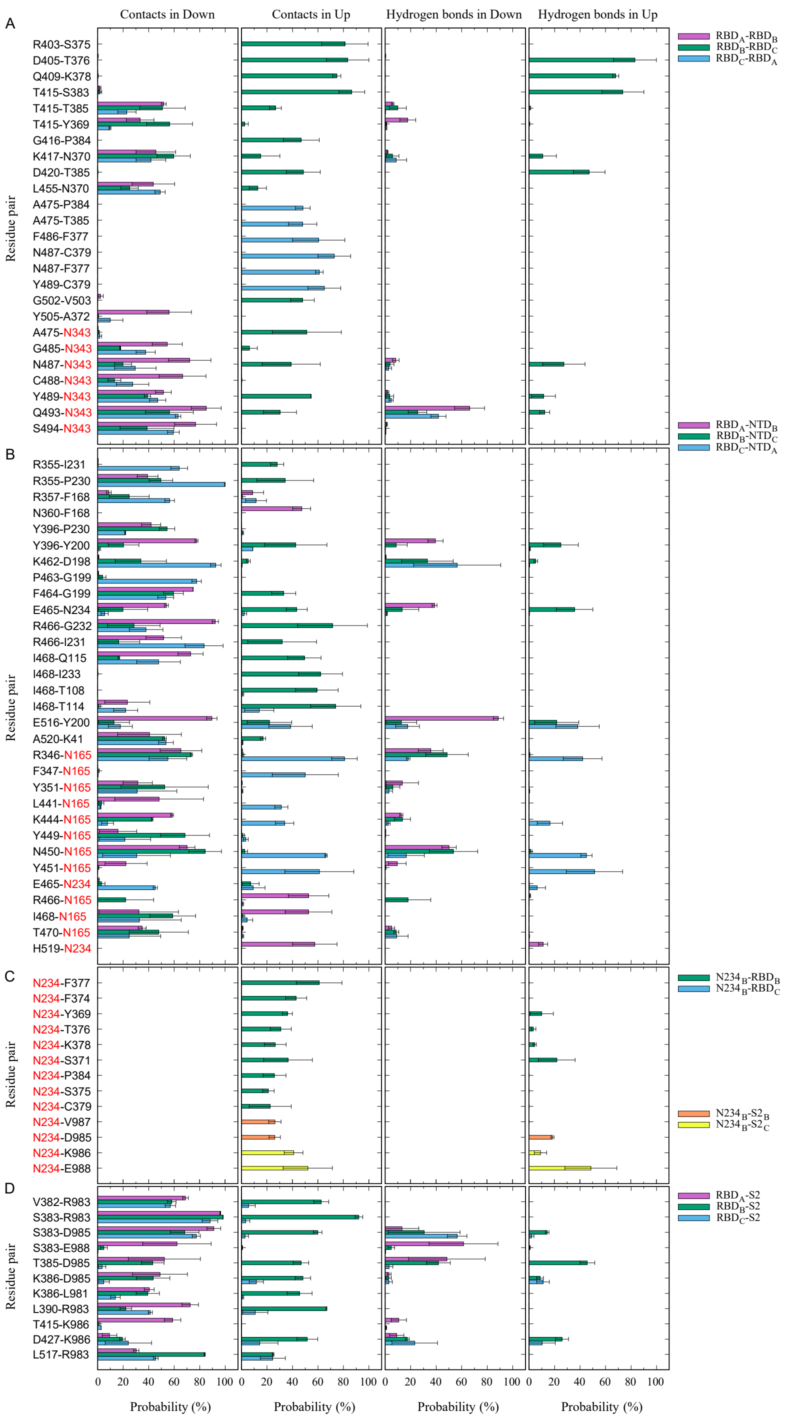

Figure S6. The interdomain contacts and hydrogen bonds in MD2_Down and MD2_Up. (A) Those interactions between different RBDs: RBD_A-RBD_B (purple), RBD_B-RBD_C (green), RBD_C-RBD_A

(light blue), (B) those between RBD and NTD: RBD_A-NTD_B (purple), RBD_B-NTD_C (green), RBD_C-NTD_A (light blue), (C) those between N234 and RBDs/S2: $N234_B$-RBD_B (green), $N234_B$-RBD_C (light blue), $N234_B$-$S2_B$ (orange), $N234_B$-$S2_C$ (yellow), (D) those between RBD and S2: RBD_A-S2 (purple), RBD_B-S2 (green), RBD_C-S2 (light blue). Red characters mean the glycosylated amino-acid residues. In the analysis, a contact is defined when the minimum residue distance is shorter than 2.5 Å. A hydrogen bond is decided if the D…A (donor…acceptor) distance is shorter than 3.4 Å, the DH…A angle is smaller than 120°, and HD…A angle is greater than 30°. A 200-ns trajectory is divided into two 100-ns trajectories and the average numbers of contacts and hydrogen bonds are shown as bars. The maximum and minimum numbers are shown in error bars. Only the residue pairs that have more than 45% (for panels A, B, and D) or 20 % (for panel C) of contacts in either MD2_Down or MD2_Up are shown.

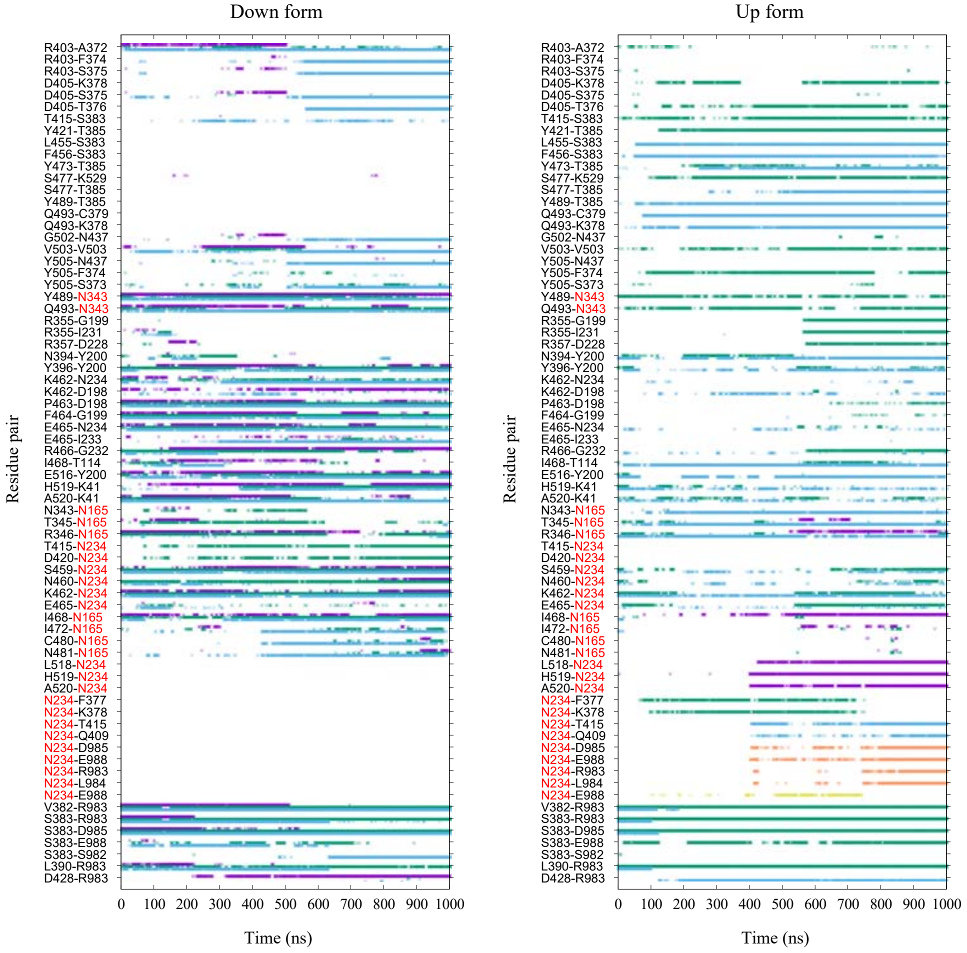

Figure S7. Time series of inter-domain contacts in MD1_Down (left) and MD1_Up (right). The color definition for dots is same as in Figure 3**.** Black characters mean the amino-acid residues, and red characters mean the glycan part of the glycosylated amino-acid residues.

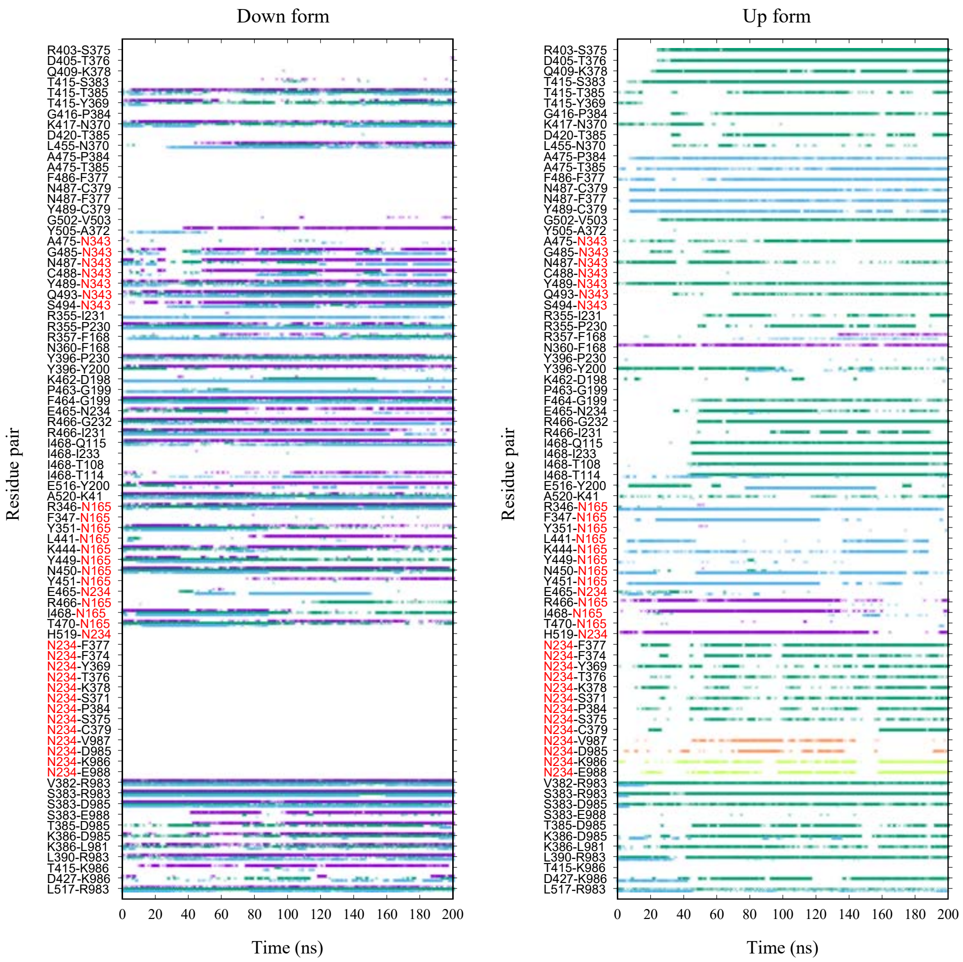

Figure S8. Time series of inter-domain contacts in MD2_Down (left) and MD2_Up (right). The color definition for dots is same as in Figure S5**.** Black characters mean the amino-acid residues, and red characters mean the glycan part of the glycosylated amino-acid residues.

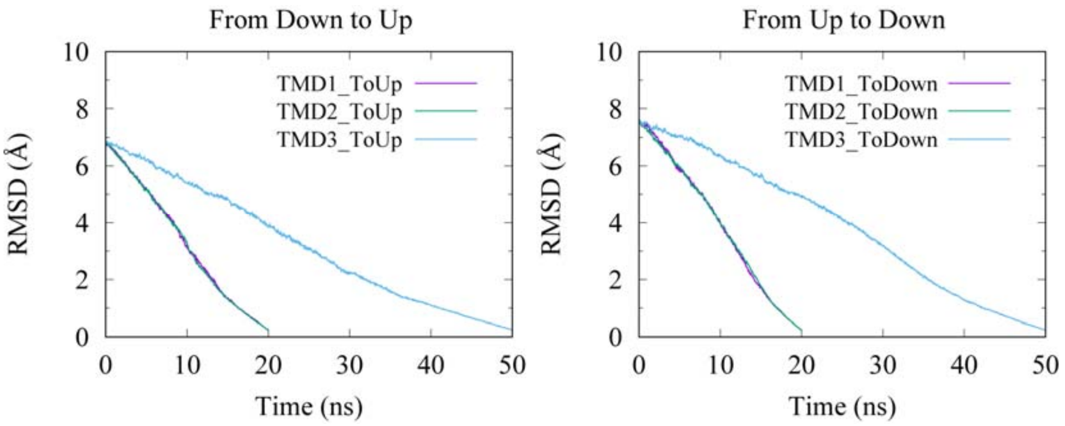

Figure S9. Time series of RMSD in TMD simulations from Down to Up (left) and from Up to Down (right). The RMSD was calculated using the Cα atoms of S1 and S2 subunits in the cryo-EM structure of the Up (PDB: 6VSB) or Down form (PDB: 6VXX).

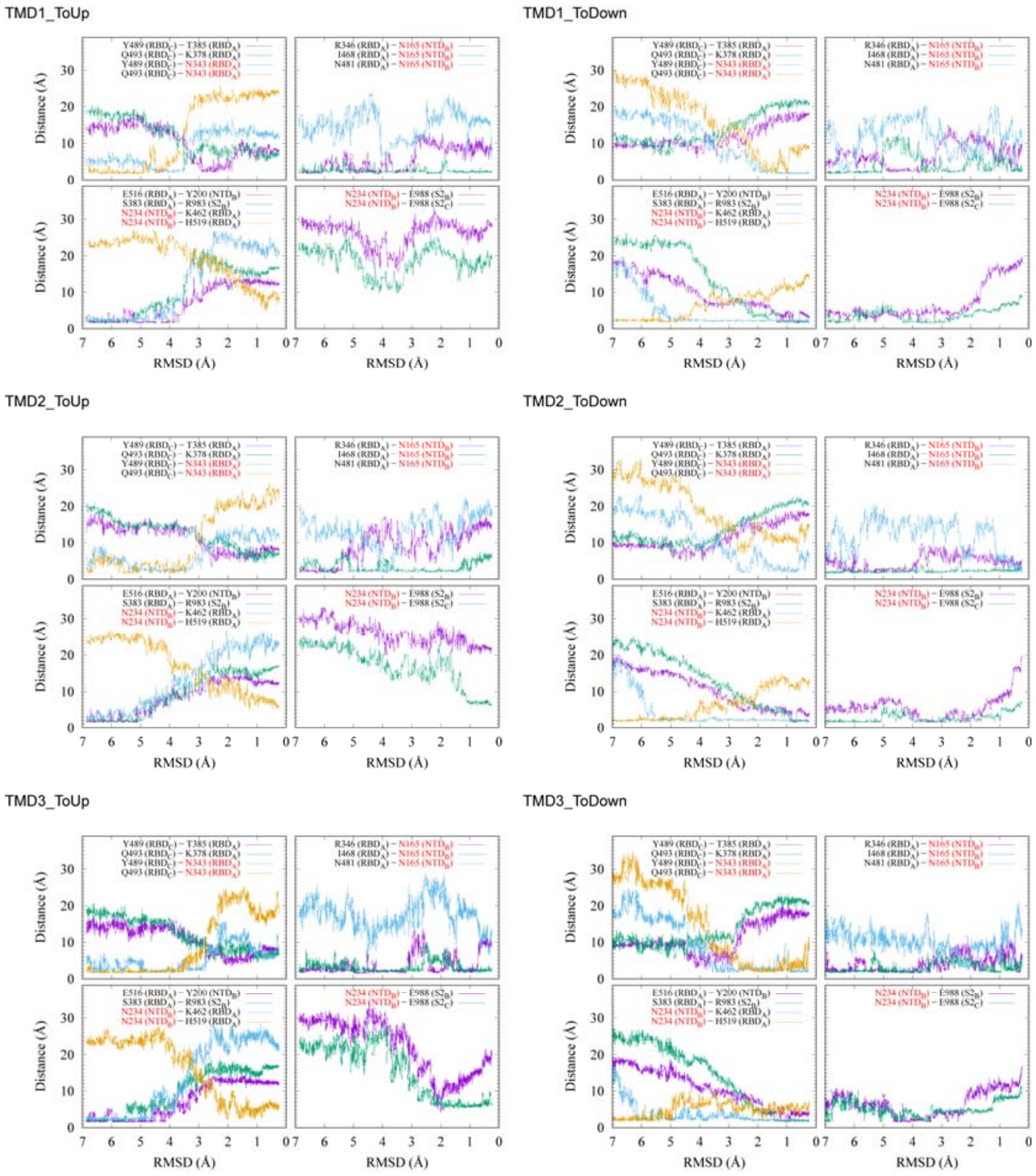

Figure S10. The minimum distances of the inter-domain amino-acid residue pairs and residue-glycan pairs in the three TMD_ToUp (left panels) and three TMD_ToDown (right panels) simulations. Red characters mean the glycosylated amino-acid residues. RMSD in TMD simulations is measured using the cryo-EM structure in the Up or Down form as a reference.
